# Estimating the effects of policies on infertility prevalence worldwide

**DOI:** 10.1186/s12889-022-13802-9

**Published:** 2022-07-19

**Authors:** Xiaochen Zhang, Quanquan Guan, Qiurun Yu, Wenwen Xiao, Ziyu Chen, Chao Dong, Siting Deng, Yin Zhuang, Yankai Xia

**Affiliations:** 1 State Key Laboratory of Reproductive Medicine, Center for Global Health, School of Public Health, Nanjing Medical University, No.101 Longmian Road, Nanjing, 211166 China; 2 Key Laboratory of Modern Toxicology of Ministry of Education, School of Public Health, Nanjing Medical University, Nanjing, 211166 China

**Keywords:** Infertility, Public Health, Health Policy, Health Insurance

## Abstract

**Background:**

Infertility has troubled millions of people worldwide while always being an ignored issue. The high cost of treatment or lack of services placed a barrier to the alleviation of infertility status. Governments play a significant role to promote infertility-related policies for better access to infertility services and comprehensive supports for infertile people.

**Methods:**

Data of infertility status indicators and infertility-related policies in ten representative countries were collected. An infertility-related policy system was established, then classification and quantification were processed according to specific criteria, and different policy implementation patterns were identified. The effectiveness of specific infertility-related policy and various patterns on infertility prevalence relief between 1990 and 2017 were evaluated via generalized linear models and analyses of covariance for the first time.

**Results:**

Economic support policies would be less prioritized compared with social security policies, while economic support policy had a significant positive role in the decline of female infertility prevalence (β = -2·16, *p* = 0·042). In detail, insurance coverage and economic reward policies were crucial (β = -3·31, *p* = 0·031; β = -4·10, *p* = 0·025) with adjusted with covariates. The effect of economic support-oriented pattern was relatively better than other patterns for both male and female infertility prevalence relief. Nevertheless, the effectiveness of gradual-promotion pattern seemed preferable for male infertility prevalence relief while was similar with simultaneous-promotion pattern for females.

**Conclusions:**

Our data-driven analysis revealed that insurance coverage and economic reward policies played the pivotal role in moderation of female infertility status. Economic support-oriented pattern and gradual-promotion pattern were preferable when promoting infertility-related policies.

**Supplementary Information:**

The online version contains supplementary material available at 10.1186/s12889-022-13802-9.

## Background

Infertility, defined as the inability to conceive a child after one year of intercourse without contraception, troubled millions of people worldwide [1]. It exerts damaging effects on individual physical and psychological health and national fertility capability, causing burdens of healthcare costs and the aging of the population [2]. In the context of diverse population problems, alleviating infertility has been an urgent issue that all countries face. With babies born through *in vitro* fertilization (IVF) for the first time in 1978, there is a trend of enlargement for infertility treatments. Technically, it provided solutions for individuals to fertility problems by treatment, while more concerns were called out for prevention and early detection of infertility [3]. In recent years, the World Health Organization (WHO) has paid more attention to “prevention” as a core policy to relieve the global burden of infertility and promoted a tertiary care level approach for infertility [4].

In addition to genetics, other factors such as sexually transmitted infections, smoking, delayed childbearing and unsafe abortion increased the risk of infertility, which were alterable by policies and interventions [1]. Compared with individuals, governments have more choices including achieving primary prevention of infertility by advocating healthy lifestyles and creating a social atmosphere conducive to fertility [5, 6]. Governments can also rely on secondary prevention, by launching early diagnosis and treatment methods to benefit fertility recovery [3]. Moreover, appropriate financial support for fertility treatment was proved to represent sound fiscal policy that benefits equities in access to care [7]. Thus, policies that directly or indirectly benefit the infertile population are conducive to achieving tertiary prevention of infertility. Governments play a significant role in promoting these infertility-related policies for better access to infertility services and comprehensive supports for them.

Recent studies were concerned about the significance and investments of some infertility-related policies, which were incomplete. Iris G. Insogna et al [8] found the unequal gaps in infertility items through the summary of medical insurance coverage in the United States. Meanwhile, other aspects like service package for infertile populations were not considered. In another study, Morshed-Behbahani et al. [9] revealed that less financial protection policy in lower middle-income countries. However, the effectiveness of particular policies on the decline in infertility prevalence still lacked evidence.

As more data was accessible, we can conclude exhaustive infertility-related policies in various countries and gradually understand the effects of individual policies. Due to the different strategies for implementing policies (e.g., the year of release, the degree of intervention) in various countries, we can classify these differences into various policy implementation patterns and compare their benefits. Our study could encourage governments to efficiently improve infertility status by focusing on the most effective infertility-related policies and appropriate implementation patterns while issuing new guidelines on the policies.

## Methods

### Source of data

**Fig. 1:**
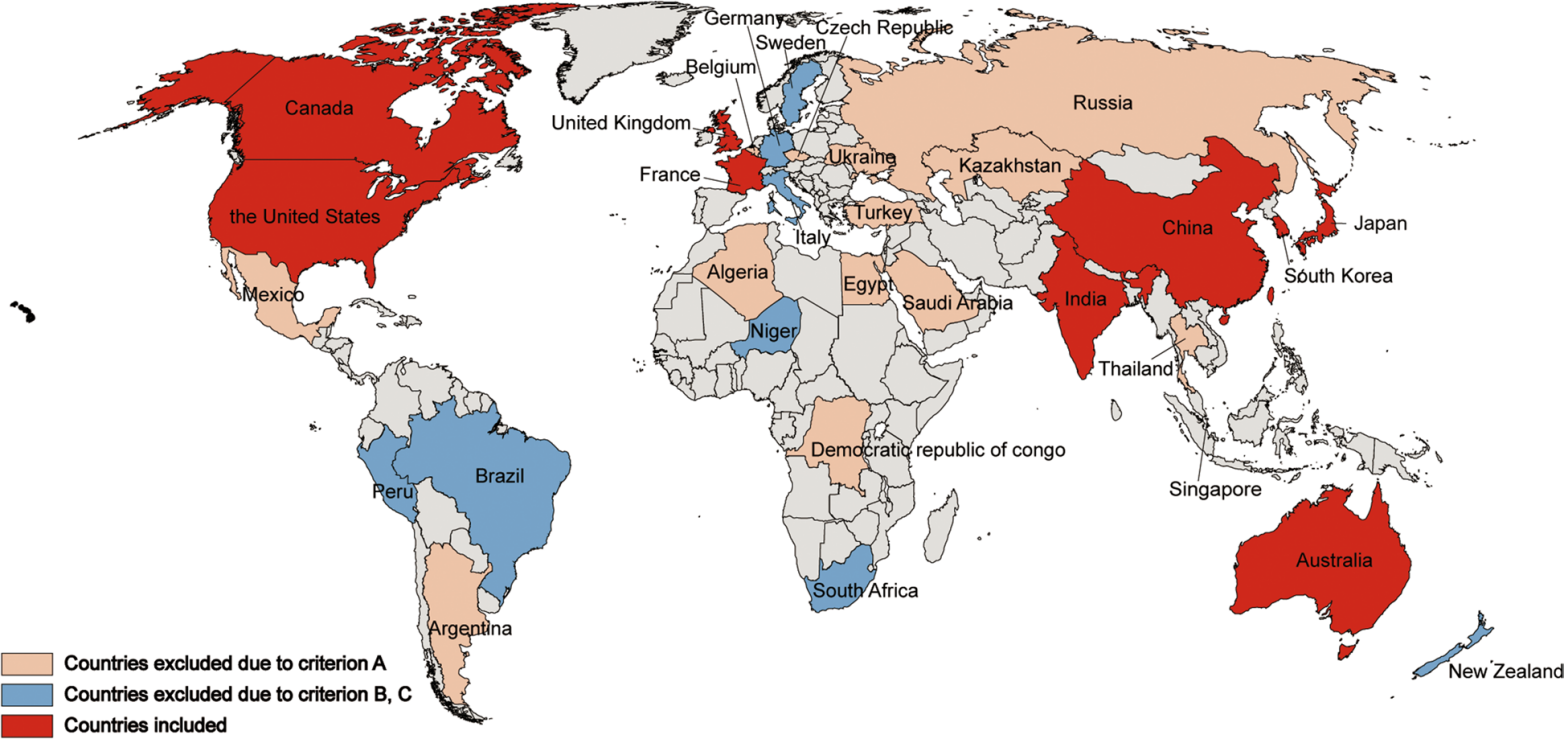
Selection procedure of included countries. Criterion **A**: Sufficient searchable information; criterion **B**: Unlimited access to documents; criterion **C**: Documents in Chinese or English

After preliminary search, over 30 continental-representative countries were initially selected according to geographical location, social culture, and income level. Eventually, ten countries (Australia, Canada, China, France, India, Japan, Singapore, South Korea, the United States, and United Kingdom) were included in this study based on the following criteria (Fig. 1):

A. Sufficient searchable information;
B. Unlimited access to documents;
C. Documents in Chinese or English.

Infertility prevalence data were searched from databases (CNKI, Weipu Journals, PubMed, Science Direct, Web of Science, Google Scholar) and government official websites. Other infertility-related indicators, including total fertility rate (TFR), male first marriage age, female first marriage age, and first childbearing age were searched from national databases and institutional websites (WHO official website, World Bank official website). National development indicators containing economic development data and existing infertility-related medical volume data were obtained from WHO, World Bank, and other research. Keywords for infertility prevalence were infertility rate, infertility prevalence, assisted reproductive technology, *in vitro* fertilization, and childlessness; for others were consistent with names of indicators.

### Classification of infertility-related policies

Three researchers screened online, available, and authoritative infertility-related policies independently and concluded them after a double-blind check. At first, the study pooled over 300 documents released in recent 30 years and finally included 265 records after excluding those irrelevant policy documents. Combining the scopes (prevention, treatment, and supportive care) of universal health coverage (UHC) with the objectives of the non-medical policy, [9] we defined the ESSTR system with five dimensions. Specifically, the ESSTR system refers to a classification method of infertility-related policies into five categories: Economic support, Social security, Service package, Technology development guarantee, and Reproductive health protection with a total of ten indexes (Table 1).

**Table 1:** Dimension, index, consensus definition and example of ESSTR system

**Dimensions and index**	**Consensus definition**	**Example**
**Economic support**		
Insurance coverage	Covering costs of infertility examinations or treatments.	Infertility examinations: semen analysis, genetic testing, clinical examinations, etc.; Infertility treatments: assisted reproductive technology (artificial insemination, IVF and its derivative technology), etc.
Financial assistance	Financial assistance from government and non-profit organizations for people seeking for assisted reproductive help.	Financial subsidies or allowance for the cost of infertility treatments that were not covered in insurance, etc.
Economic reward	Economic reward from government for fertility.	Baby bonus, second child reward, multiple children reward, etc.
**Social security**		
Infertility diagnosis and treatment leave security	Policies to guarantee legal infertility diagnosis and treatment leave for people seeking for assisted reproduction assistance.	Paid or unpaid infertility examination leave, infertility treatment leave, etc.
Parental leave security	Policies to guarantee legal parental leave for parents.	Paid or unpaid parental leave for fathers and mothers; protecting working women during marriage, pregnancy and childbirth; Eliminating worries about not being able to balance work and family, etc.
Child care and education security	Policies to guarantee the basic childcare and educational needs of children.	Children’s enrollment, childcare facilities, compulsory education, etc.
Life security	Policies to guarantee the basic living needs of children.	Housing security, living security, family welfare, etc.
**Service package**		
Maternal and child health service	Policies to improve the level of maternal and child health services and health.	Prenatal, pregnancy, and postpartum health care services, etc.
**Technology development guarantee**		
Assisted reproductive technology development	Policies to support the maintenance and expansion of assisted reproductive technology and research progress.	Assisted reproductive technology research funding, talent introduction policy, etc.
**Reproductive health protection**		
Reproductive health education and protection	Policies to promote public reproductive health and quality.	Sex education, sexually transmitted disease prevention and education, etc.

*Abbreviation*: *ESSTR* Economic support, Social security, Service package, Technology development guarantee, and Reproductive health protection, *IVF In vitro* fertilization

### Quantification of infertility-related policies in implementation paces

To explore the implementation paces of infertility-related policies in selected countries, three stages were classified according to the number, degree of intervention, and specific content of infertility-related policies:

1. Stage I, no intervention: no relevant policy or the content of policies negative or neutral,
2. Stage II, partial intervention: relevant policy but not complete or the content of policies neutral and positive,
3. Stage III, active and comprehensive intervention: relevant policy more complete or the content of policies more active.

Policies without a clear release time were not included in the grading process. The basis of the policy grading was generated through an inclusive consensus-based process and shown in Additional file 1, Table S1. To better describe the characteristics of dynamic changes, these countries were clustered into two patterns in terms of each index’s implementation pace, which were gradual-promotion and simultaneous-promotion patterns. All records with clear resource and quantification were shown in Additional file 1, Appendix 2.

### Quantification of infertility-related policies in policy orientation

The process of quantification of infertility-related policies in policy orientation was discussed by independent experts. Based on the grading results between 1990 and 2017, the annual investment (AI) of infertility-related policies was scored so that Stage I obtained 0, Stage II obtained 1, and Stage III obtained 2. On a percentage basis, the Cumulative Investment Index (CII) of infertility-related policies was finally built to evaluate the cumulative promotion degree of each category of policy between 1990 and 2017. The formula was as follows:

$$\mathrm{CII}=\frac{\sum \mathrm{AI}}{\mathrm{Theoretical maximum of CII}}*100\%$$

With the comparison of stages and scores of economic support and social security policies, countries were clustered into economic support-oriented, social security-oriented, and balanced patterns. Horizontal comparisons among countries and infertility-related policies were conducted from the perspective of implementation paces and policy orientation.

### Evaluated effects of infertility-related policies on infertility

Infertility-related policies included in statistical analysis were quantified by performing the rank conversion on the difference of grading results between 1990 and 2017. Due to the inconsistency of survey scales, populations, and methods in various infertility prevalence studies, it was not comparable to directly conclude the results of different studies. Estimated age-standardized infertility prevalence changes from 1990-2017 [10] (Additional file 1, Table S2) were used to reflect infertility trends in selected countries. Meanwhile, the prevalence of primary infertility among all women in 1990 worldwide, estimated by Mascarenhas M N et al. [1] was included as the baseline in this study to adjust the effect of various policies (Additional file 1, Table S3).

The relationship between infertility-related policies and infertility was particularly complex, and the actual effectiveness of each policy was affected by a number of factors. In detail, couples postponing childbirth were more likely to take the risk of infertility. Thus, infertility baseline, infertility-related and national development indicators were considered as potential covariates in analyses. Generalized linear models were applied for analyses of infertility-related policies and potential covariates on infertility. Spearman correlation analyses were used for final selection of covariates. More accurate effects of infertility-related policies on infertility were assessed with generalized linear models adjusted with selected covariates. For comparison of different patterns in implementation paces and policy orientation, analyses of covariance were conducted. With adjusted by infertility prevalence baseline, means of infertility prevalence changes were used to compare the effects of different patterns on infertility relief. R 3.6.2 and SPSS 26 were used for statistical analyses. All analyses were two-sided tests and the significant level was 0·05.

## Results

### National development in selected countries

Ten countries in this study were international representative and covering comprehensive races, different income levels and development status (Additional file 1, Table S4). Generally, infertility prevalence was 12-24% in the past decade. Total fertility rates (TFR) in most surveyed countries were lower than the replacement level of 2·1 except India (2·22). TFR of South Korea was as low as 0·98, which ranked the last among ten countries. The average age at first marriage and childbirth was generally delayed in high-income countries. In terms of assisted reproductive technology (ART) development, the high technological and economic level revealed no complete consistency with the large-scale assisted reproductive market, as the small number of ART institutions or IVF cycles annually in some developed countries (Singapore, the United Kingdom, and Canada).

### Infertility in recent decades

Forty studies of infertility prevalence around ten countries were included (Additional file 1, Table S5). Three main research methods including cross-sectional, prospective study, and national estimates were applied to estimate infertility prevalence, causing small range variations of results. This can be seen in the example of China, where estimations of infertility prevalence range from 13·6 to 20% around 2011. Studies with the same method reported matching infertility prevalence in each country. For instance, two independent cross-sectional studies in 2001 in China concluded similar results (17·13 and 18·00%) despite inconsistent research scales. Those researches spanned 40 years, and the prevalence of infertility exhibited a rapid rise in recent years generally. It was confirmed that infertility prevalence in different age groups varied and increased with age by cohort study. For gender, the prevalence of male infertility was higher than that of female in the United Kingdom in 2010-2012. Although it was considered as severe as female infertility, only a few studies focused on estimates of male infertility prevalence.

### Implementation and investment of infertility-related policies

**Fig. 2:**
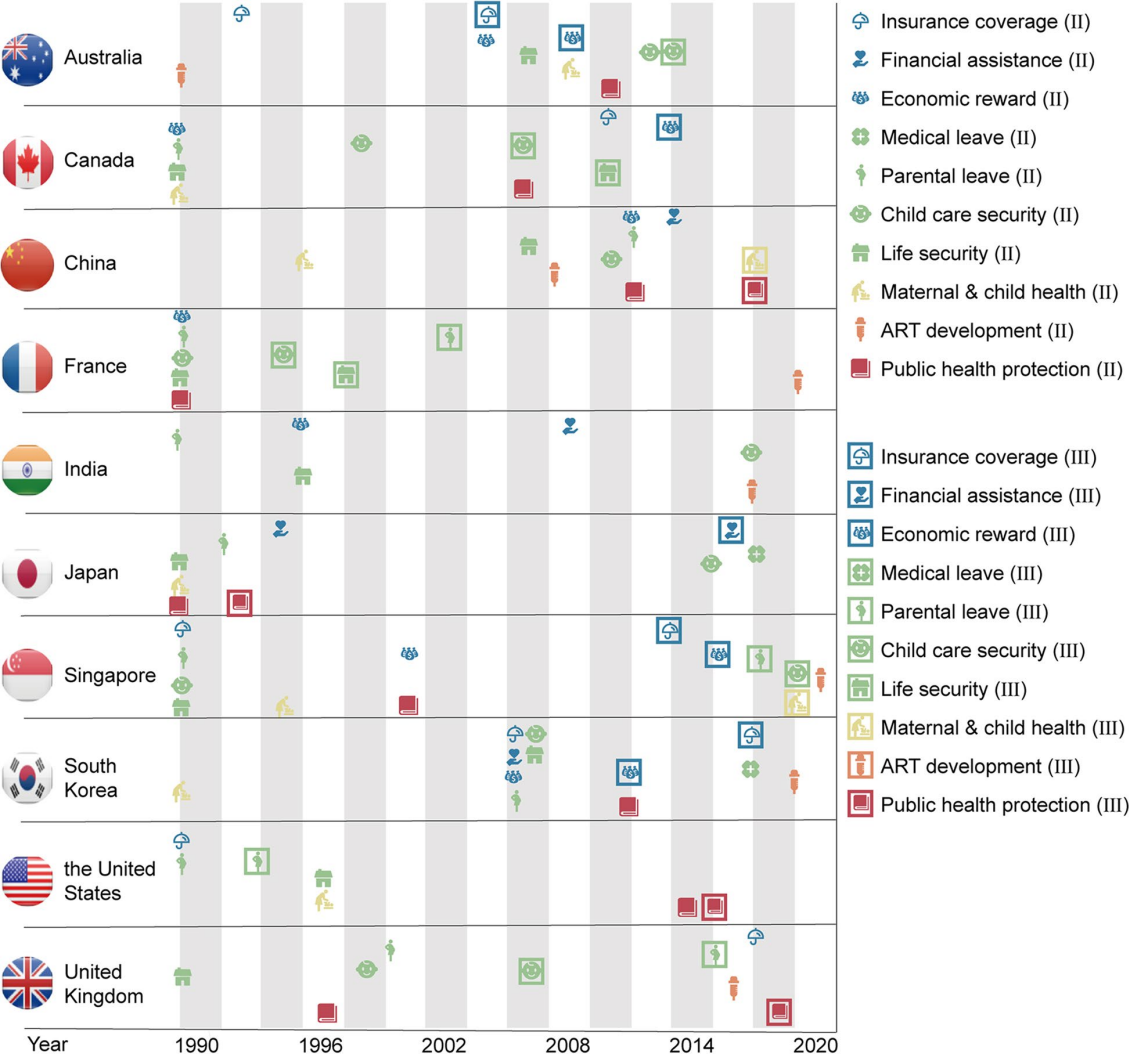
Landscape of infertility-related policies development in ten countries

**Fig. 3:**
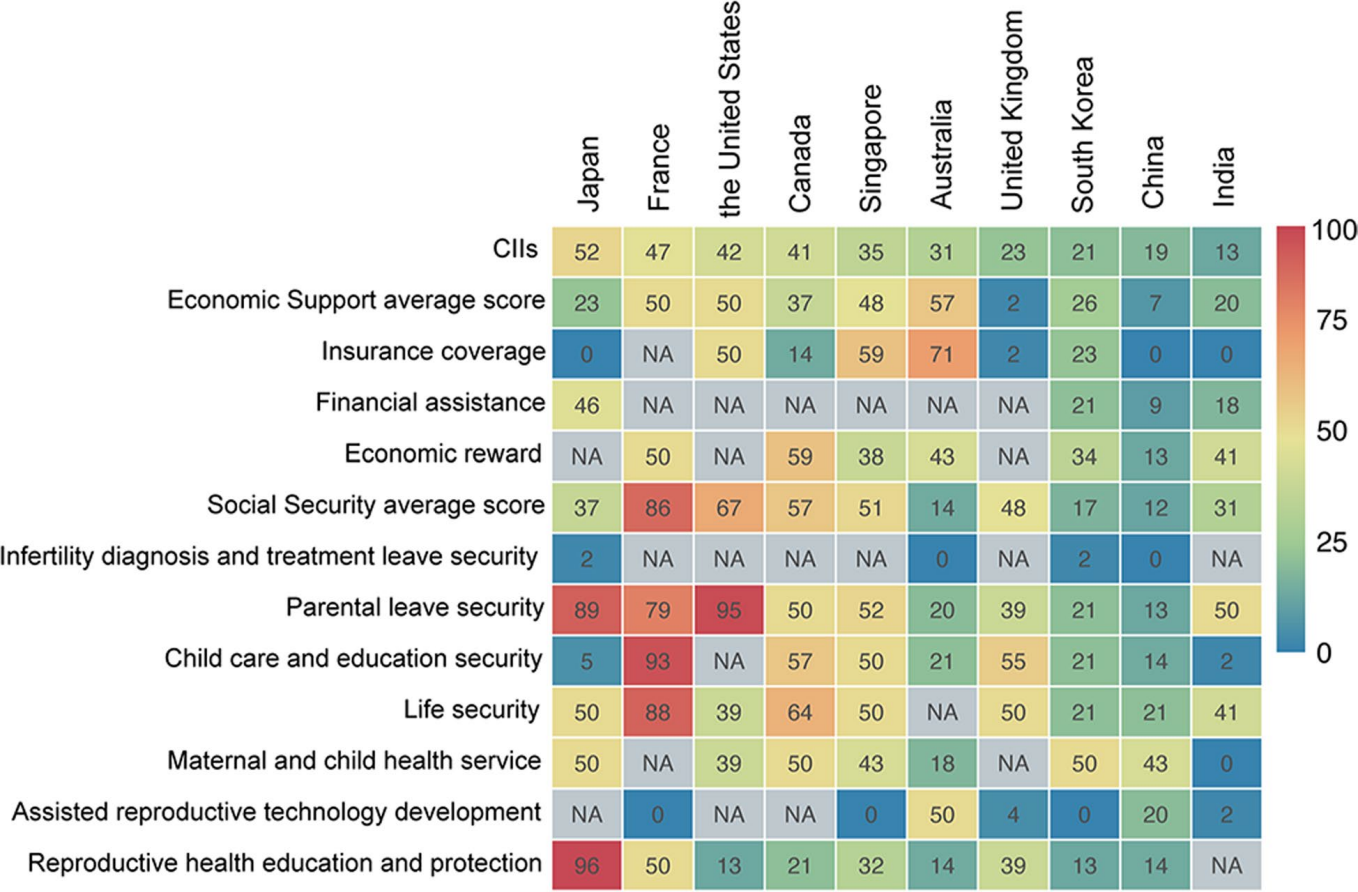
Elaboration of CIIs and specific infertility-related policy scores in ten countries. CII: Cumulative Investment Index

Implementation and investment of infertility-related policies varied among included countries. Generally, more infertility-related policy items were in Stage III in high-income countries than lower-middle and upper-middle income countries (Fig. 2). We used CII to evaluate the overall progress of infertility-related policies implementation, and found CIIs increased with the national income level. In another word, high-income countries invested more in policies among ESSTR (Fig. 3).

The governments had a certain tendency to promote infertility-related policies. Looking around ESSTR, economic support policies would be less prioritized compared with social security policies. From 1990 to 2020, a total of five countries implemented stronger policies in the economic support category while seven countries did that in social security category (Stage III). Three countries (China, India, and Japan) were still under Stage I with no relevant insurance coverage policies (Fig. 2). CIIs of social security policies were higher than economic support policies in ten countries. Although reproductive health education and protection policy scores were extremely high in Japan, it was not universal in other countries. For this feature, ten countries were clustered into three policy orientation patterns. South Korea, Singapore, and Australia preferred infertility-related economic support policy, while France, the United Kingdom, and the United States were social security-oriented patterns. Other four countries including China, India, Japan and Canada were basically balanced between economic support and social security (Fig. 3).

Figure 2 also showed that the speed of implementing infertility-related policies varied across countries. On the one hand, China, India, Japan, the United Kingdom, Australia, and Canada followed a gradual-promotion pattern. On the other hand, South Korea, Singapore, France, and the United States had simultaneous-promotion patterns. Notably, countries with the same policy orientation pattern had different implementation paces. Singapore and Australia were both the economic support-oriented pattern while diverse in implementation paces.

### Effects comparison of infertility-related policies and patterns on infertility prevalence

The trends of age-standardized infertility prevalence change estimated by Sun H *et al.* from 1990 to 2017 (Additional file 1, Table S2) were approximately consistent with the results of forty studies. Infertility prevalence in India, France, and the United States increased both in males and females, and decreased changes were observed in Singapore, United Kingdom, and Australia. Besides, age-standardized male infertility prevalence dropped from 1990 to 2017 in China, Japan and Canada, and female infertility prevalence dropped in South Korea.

#### Male infertility

Our model enabled us to estimate the individual effect of each infertility-related policy, expressed as a value change on infertility prevalence. As Table 2 showed, model 1 displayed the potential positive effects of four policy categories while having no statistical significance. Adjusted with covariates of infertility baseline, first childbearing age, and per capita GDP, parental leave security policy might have a certain positive effect on alleviating infertility although not statistically significant (β = -0·79, *p* = 0·096).

**Table 2:** Analyses of policies and age-standardized infertility prevalence from 1990-2017

	Model 1^a^								Model 2^b^							
	**Male infertility**				**Female infertility**				**Male infertility**				**Female infertility**			
	β	95% CI		*p*	β	95% CI		*p*	β	95% CI		*p*	β^c^	95% CI		*p*
**Economic support**	-0·79	-1·88	0·29	0·189	-2·81	-4·77	-0·85	**0·023**	-0·54	-1·38	0·31	0·268	-2·16	-3·72	-0·60	**0·042**
Insurance coverage	-0·60	-1·87	0·67	0·383	-3·19	-5·31	-1·07	**0·021**	0·22	-1·26	1·70	0·785	-3·31	-5·30	-1·33	**0·031**
Financial assistance^c^	··	··	··	··	··	··	··	··	··	··	··	··	··	··	··	··
Economic reward	-1·78	-4·49	0·94	0·256	-6·72	-11·23	-2·21	**0·033**	-0·40	-2·99	2·19	0·791	-4·10	-5·38	-2·81	**0·025**
**Social security**	-0·75	-2·29	0·80	0·371	-0·89	-4·54	2·77	0·646	-0·52	-1·54	0·49	0·360	-0·20	-3·03	2·63	0·895
Infertility diagnosis and treatment leave security^c^	··	··	··	··	··	··	··	··	··	··	··	··	··	··	··	··
Parental leave security	-1·11	-2·24	0·03	0·092	-1·19	-4·19	1·81	0·459	-0·79	-1·54	-0·03	0·096	0·16	-2·43	2·75	0·906
Child care and education security	-0·51	-2·06	1·04	0·536	-0·34	-3·95	3·27	0·859	-0·40	-1·31	0·51	0·437	-0·08	-2·80	2·64	0·955
Life security	1·69	-0·83	4·20	0·231	2·87	-3·26	9·00	0·389	1·16	-0·17	2·49	0·163	0·85	-4·62	6·32	0·776
**Service package**	-0·75	-2·32	0·81	0·384	-1·08	-4·95	2·79	0·604	-0·58	-1·61	0·44	0·347	-1·31	-4·22	1·61	0·444
**Technology development guarantee**	0·57	-2·01	3·15	0·683	3·50	-1·91	8·90	0·261	-1·54	-4·67	1·58	0·435	-0·15	-7·48	7·17	0·971
**Reproductive health protection**	-0·19	-1·04	0·65	0·666	0·71	-1·80	3·22	0·596	0·87	-1·94	3·68	0·575	0·30	-7·38	7·98	0·943

^a^Not adjusted

^b^Adjusted with infertility prevalence baseline, first childbearing age and per capita GDP

^c^Effectiveness of financial assistance, and infertility diagnosis and treatment leave security policies was not been evaluated because of missing data in most countries

*Abbreviation*: *CI* Confidence interval

**Fig. 4:**
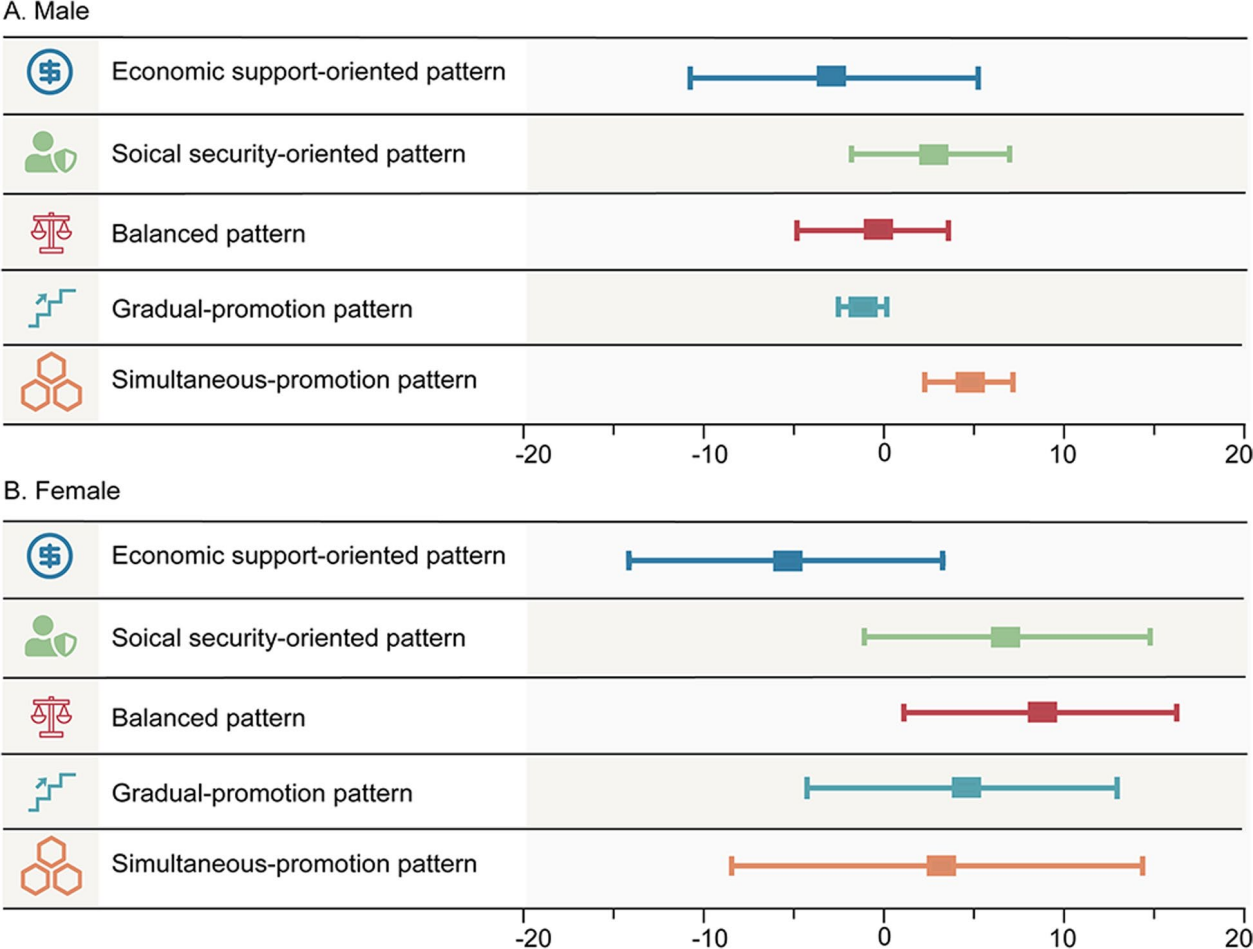
Comparison of effects of different patterns on male and female infertility prevalence, respectively

Figure 4 showed the effectiveness of policy orientation and implementation pace patterns that were presented as means of change values. On average, after adjusting the infertility baseline, the effect of economic support-oriented pattern (-2·95, [-11·05, 5·16]) was relatively better than other patterns (2·20, [-2·77, 7·17]; -0·57, [-4·99, 3·85]). Nevertheless, the effectiveness of the gradual-promotion pattern (-1·35, [-2·85, 0·14]) was preferable for male infertility relief (Additional file 1, Table S6).

#### Female infertility

Under the default model settings, economic support policy had a significant positive role on female infertility improvement (β = -2·81, *p* = 0·023). In detail, insurance coverage and economic reward policies were crucial (β = -3·19, *p* = 0·021; β = -6·72, *p* = 0·033). With adjusted with covariates, effectiveness of infertility-related economic security policy, especially insurance coverage and economic reward policies, had been proved (β = -2·16, *p* = 0·042; β = -3·31, *p* = 0·031; β = -4·10, *p* = 0·025) (Table 2).

Similar to male, the change in female infertility prevalence of the economic support-oriented countries (-5·42, [-14·04, 3·20]) was larger than that of the other two types of countries (6·71, [-1·50, 14·92]; 8·81, [1·37, 16·25]). Gradual-promotion pattern and simultaneous-promotion pattern were similar on female infertility prevalence relief (4·33, [-4·32, 12·98]; 3·28, [-7·47, 14·03]) (Additional file 1, Table S6).

## Discussion

We used a data-driven approach to evaluate the effects of infertility-related policies on the relief of infertility in ten countries between 1990 and 2017. We included research objects in view of geographical representation and evidence-based exclusion criteria that covered comprehensive species, three gradations of income levels, and different national development statuses. As a consequence, the results fully considered the differences generated by various races and economic development levels and were representative around the world. Our model first proved that insurance coverage and economic reward policies were related to a clear reduction in female infertility prevalence. In addition, we found that the economic support-orientation pattern outperformed others, and the gradual-promotion pattern was preferable for male infertility prevalence improvement.

Our results revealed the continuously severe infertility problem worldwide and common late marriage and childbirth in high-income countries. Combined with current reports, the infertility prevalence was 12-24% in selected countries. The frequency of involuntary infertility in a nationally representative population was estimated as high as 24% in France in 2007-2008, [11] which was above the global infertility prevalence reported by WHO (about 15%), warning about the critical infertility situation we face [12]. As a disease with complex etiology and various factors, infertility was influenced not only by a number of psychological and pathophysiological factors in both partners of a couple, but also by multiple social, economic, gestational age, and physiological factors changing over time. In developed countries, socioeconomic advantages with high-income level and education are associated with fecund postponement and infertility [13, 14]. A national survey study of 850 US surgeons reported that female surgeons were more likely to delay pregnancy because of work, leading to increased medical risks of infertility [15]. Noticeably, increased environmental health pressure, unwanted pregnancy, and unsafe abortion also exacerbated the infertile crisis in developing countries, although the average fertile age is relatively low [16]. Meanwhile, limited access to reliable infertility diagnosis and treatment aggravates the infertility problem in those countries [17]. What’s worse, infertility is a low-priority issue in countries with high fertility rates and limited health resources, considered as a natural method to overpopulation by those governments [18]. Therefore, encouraging populations to give birth at the optimal age and raising awareness of reproductive health through appropriate policies are urgent issues over the period of infertility alleviation.

The establishment of ESSTR system expanded the theoretical framework in infertility policy-making. Economic reward policy and four policies in the Social Security dimension stimulated public willingness to have children [19]. The effectiveness of the economic reward policy for the decrease of female infertility prevalence was proved in our study, which revealed that the additional effects of fertility policies on infertility relief were underestimated. On the one hand, infertility caused by late marriage and childbirth could be avoided to some extent. On the other hand, comprehensive support policies might encourage infertile people to seek medical treatment. Other fertility supporting policies for children’s enrollment, childcare facilities, compulsory education & housing security, living security, and family welfare provided backup force and solved the back-end problem, despite no statistical significance in our study. Furthermore, fertility supporting policies are related to the increase of national fecundity level, that could coordinate the resolution of population structure issues such as the aging of population, and promote sustainable development [20]. The significance of reproductive health care and public education was shown in other studies. Accessible maternal & child health service and sexual education decrease the risk of reproductive diseases, which have positive consequences for infertility prevention [21].

We found a large effect of insurance coverage policy for female infertility status relief, which was remarkably robust across different model structures. Taking infertility treatments into insurance coverage encourages patients to seek assisted reproductive help at a younger age, which is a significant factor in the live-birth rate of the IVF cycle [22]. Andrew D. A. C. Smith et al [23] reported that female patients younger than 40 years experienced the highest live-birth rate for the first cycle than women aged 40-42 years and older than 42 years. Besides, most infertile patients undergo IVF cycles several times during their successful treatment. It was illustrated that female patients with insurance coverage were more likely to experience IVF again, which means the increasing cumulative live-birth rate compared with single IVF cycle, and had a higher cumulative probability of live-birth than patients who paid for IVF personally [23]. In low- and middle-income countries, limited ART treatments are provided for infertile people because of limited resources and high treatment costs. However, a South African case study estimating the government public economic benefits attributed to investing in ART suggested that funding for IVF may create positive economic benefits and promote the sustainability of health systems [24].

Theories of the Policy Cycle emphasized the core position of policy formulation and implementation, and how to promote policies efficiently is a question worthy of discussion [25]. Policies of relieving infertility status cover associated aspects that containing insurance coverage, government funding, service optimization, clinic and laboratory supervision, and public health guidance [3]. The main difficulty is how to find the most cost-effective policy in the context of limited resources. It is also significant to regulate assisted reproductive technologies and avoid over-regulating [26]. Our study showed that insurance coverage investment was at a low level in most surveyed countries. Gender inequality also exists in that some regions take female infertility items into insurance coverage. In contrast, male items do not, which hinders the goal-achievement of universal health coverage (UHC) [27]. The positive association between national income level and UHC performance causes difficulties in implementing an infertility insurance policy. In comparison, governments could improve both financial protection and service coverage within the capacity to achieve the goal of UHC [28].

Classification and quantification of infertility-related policies in various countries make it possible to identify different patterns of implementation paces and policy orientation. Combined with results for male and female infertility improvement, the overall effect of the economic support-oriented pattern was better than that of the social security-oriented and balanced pattern. It prompted to prioritize the implementation of insurance coverage and/or birth reward policies within the limits of national power. Compared with other public policy models, incrementalism, which was proposed by Charles E. Lindblom, is more easily accepted by policymakers because of the consistency of actual policies, continuity of current plans, and the difficulty of technology [29]. Similar conclusions were also reflected in the policy implementation paces. Our research suggested that policy implementation can be more progressive when alleviating the problem of infertility. For countries with large population bases and limited economic resources, a gradual-promotion pattern could be considered more cost-effective than a simultaneous-promotion pattern. Implementing several policies gradually was low-cost, especially in countries with a large population base and certain restrictions on economic investment. Otherwise, despite similar effects on female, a gradual-promotion pattern was preferable for the relief of male infertility.

There were still several limitations in this study. Due to restrictions on the language or access to the data, some documents lacked specific years and were not included when grading and scoring in order to avoid bias, and thus only ten countries were included in our study. Additional studies with more adequate data will be needed to develop a full picture of infertility-related policies quantitative research. On the other hand, it is recognized that precise numbers of the infertile population are difficult to be estimated, and different global studies concluded inconsistent results [30]. In this study, age-standard infertility prevalence estimated by Sun H et al. [10] was used for statistical analyses.

## Conclusions

Governments around the world seek to relieve infertility while minimizing the social and economic costs of their policy implementations. Our data-driven analysis revealed that insurance coverage and economic reward policies played the pivotal role in moderation of female infertility status. Economic support-oriented pattern and gradual-promotion pattern were preferable when promoting infertility-related policies. These results enriched infertility policy research, and provided theoretical basis and practical guidance for government decision-making that gradually promoting economic support policies could be prioritized; however, our estimates should not be taken as the final word on infertility-related policy effectiveness.

## Supplementary Information

**Additional file 1.**

## Data Availability

All data generated or analysed during this study are included in this published article and its supplementary information files.

## Abbreviations

AI: Annual Investment
ART: Assisted Reproductive Technology
CI: Confidence Interval
CII: Cumulative Investment Index
ESSTR: Economic support, Social security, Service package, Technology development guarantee, and Reproductive health protection
GDP: Gross Domestic Product
IVF: *in vitro* Fertilization
TFR: Total Fertility Rate
UHC: Universal Health Coverage
WHO: World Health Organization
