# Supplementary material for: Estimating the effects of policies on infertility prevalence worldwide
Source: BMC Public Health. 2022 Jul 19;22:1378. doi: 10.1186/s12889-022-13802-9 (PMC9295370; doi:10.1186/s12889-022-13802-9)
Supplement: Supplementary file 1 — Additional file 1. [file 12889_2022_13802_MOESM1_ESM.pdf]

Contents

Table S1. The basis of the policy classification

Table S2. Age-standardized infertility prevalence in 1990-2017

Table S3. Prevalence of primary infertility among all women in 1990

Table S4. Status of infertility and national development in selected countries

Table S5. Infertility prevalence of the selected countries

Table S6. Comparison of different patterns on infertility

Appendix 1. Data source of table S4

Appendix 2. Infertility-related policies in 10 selected countries (including classification and sources). A. Australia; B. Canada; C. China; D. France; E. India; F. Japan; G. Singapore; H. South Korea; I. the United States; J. United Kingdom.

Appendix 3. Calculation process of CIIs. A. Australia; B. Canada; C. China; D. France; E. India; F. Japan; G. Singapore; H. South Korea; I. the United States; J. United Kingdom.

Table S1. The basis of the policy classification

	Grading criteria	Stage
**Dimensions and Index**		
Economic support		
Insurance coverage	No relevant policy / No coverage	
Financial assistance	No relevant policy / No assistance	
Economic reward	No relevant policy / No reward	
Social security		
Infertility diagnosis and treatment leave security	No relevant policy / No leave security	
Parental leave security	No relevant policy / No leave security	
Child care and education security	No relevant policy	I
Life security	No relevant policy	
Service package		
Maternal and child health service	No relevant policy	
Technology development guarantee		
Assisted reproductive technology development	No relevant policy	
Reproductive health protection		
Reproductive health education and protection	No relevant policy	
Economic support		
Insurance coverage	Limited examinations or treatments coverage / people / coverage ratio	
Financial assistance	Limited people / districts	
Economic reward	Limited people / districts	
Social security		II
Infertility diagnosis and treatment leave security	Moderate policy	
Parental leave security	Moderate policy	
Child care and education security	Moderate policy	

Life security	Moderate policy	
Service package		
Maternal and child health service	Moderate policy	
Technology development guarantee		
Assisted reproductive technology development	Moderate policy (e.g., Decentralizing power, simplifying processes, promoting scale development)	
Reproductive health protection		
Reproductive health education and protection	Moderate policy	
Economic support		
Insurance coverage	Extended examinations or treatments coverage / people / coverage ratio	
Financial assistance	Extended people / districts	
Economic reward	Extended people / districts	
Social security		
Infertility diagnosis and treatment leave security	Increased holiday duration / increased holiday salary	
Parental leave security	Increased holiday duration / increased holiday salary	
Child care and education security	Extended covered aspects / increased benefits	III
Life security	Extended covered aspects / increased benefits	
Service package		
Maternal and child health service	Extended covered aspects / support	
Technology development guarantee		
Assisted reproductive technology development	Complete policy	
Reproductive health protection		
Reproductive health education and protection	Extended covered aspects / support	

Table S2. Age-standardized infertility prevalence in 1990-2017

	Female				Male			
	PC	APC			PC	APC		
	Value	Value	95%CI	95%CI	Value	Value	95%CI	95%CI
**By country**								
Australia	-2·709	-0·153	-0·228	-0·078	-3·024	0·018	-0·074	0·109
Canada	3·723	0·006	-0·109	0·122	-1·317	0·023	-0·040	0·087
China	5·172	0·062	-0·007	0·131	-2·515	0·060	-0·032	0·153
France	14·736	0·520	0·367	0·674	7·023	0·380	0·267	0·493
India	29·267	0·876	0·517	1·237	14·162	0·420	0·231	0·610
Japan	9·850	0·015	-0·206	0·237	-3·366	0·120	-0·037	0·278
Singapore	-10·902	-0·466	-0·737	-0·193	-0·574	0·189	0·018	0·359
South Korea	-14·136	-0·447	-0·684	-0·211	0·017	0·112	0·036	0·187
the United States	6·358	-0·789	-1·957	0·392	0·672	-0·385	-0·628	-0·141
United Kingdom	-2·239	0·066	-0·147	0·280	-2·212	0·088	-0·014	0·189

Abbreviation: PC: percent change, APC: annual percent change, CI: confidence interval.

Table S3. Prevalence of primary infertility among all women in 1990

	Prevalence of primary infertility			Age-standardized prevalence of primary infertility		
	Estimate	Lower 95% uncertainty interval	Upper 95% uncertainty interval	Estimate	Lower 95% uncertainty interval	Upper 95% uncertainty interval
**By country**						
Australia	1·2%	0·3%	2·9%	1·2%	0·3%	2·9%
Canada	1·1%	0·4%	2·4%	1·1%	0·4%	2·4%
China	1·1%	0·8%	1·3%	1·1%	0·8%	1·3%
France	1·3%	0·6%	2·4%	1·3%	0·6%	2·4%
India	2·5%	2·1%	3·0%	2·5%	2·0%	3·0%
Japan	0·9%	0·3%	1·8%	0·9%	0·3%	1·8%
Singapore	0·9%	0·3%	1·9%	0·9%	0·3%	1·9%
South Korea	0·8%	0·4%	1·2%	0·8%	0·4%	1·2%
the United States	1·0%	0·8%	1·3%	1·0%	0·7%	1·2%
United Kingdom	1·2%	0·5%	2·2%	1·2%	0·5%	2·2%

Table S4. Status of infertility and national development in selected countries

	Year	Australia	Canada	China	France	India	Japan	Singapore	South Korea	the United States	United Kingdom
**Infertility-related data**											
Infertility prevalence (%)[a]	Current	15 (2015)	11·5-15·7 (2009-10)	12-18 (2021)	24 (2007-08)	10-14 (2018)	10-15 (2009)	14 (2013)	13·50 (2018)	10 (2019)	14 (2013)
Total fertility rate	2018	1·74	1·50	1·69	1·88	2·22	1·42	1·14	0·98	1·73	1·68
Male first marriage age (year)	2010-2016	31·2	28·7	27·1	33·7	26·0	30·5	30·4	33·9	29·2	28·7
Female first marriage age (year)	2010-2016	29·9	27·2	25·4	32·0	21·4	29·2	27·9	31·5	27·5	27·0
First childbearing age (year)	2015-2019	30·7	29·2	26·3	30·6	20·4	30·7	30·5	32·2	26·8	28·8
**Economic development data**											
National income level	2020	High	High	Upper-middle	High	Lower-middle	High	High	High	High	High
Development Status	2020	Developed	Developed	Developing	Developed	Developing	Developed	Developed	Developed	Developed	Developed
Per capita GDP ($)	2019	54907	46194	10261	40493	2104	40246	65233	31761	65118	42300
Health expenditure (% of GDP)	2020	9·2	10·6	5·2	11·3	3·5	10·9	4·4	7·6	17·1	9·6
Per capita medical and health expenditure ($)	2018	5425	4995	501	4690	73	4267	2824	2543	10624	4315
**Existing infertility-related medical volume data**											
**Average cost of IVF ($)**	2019	5200	7200	2400	4200	3238	4047	7284	1600	10000	7500
**Number of assisted reproductive institutions**	2019	100	34	523	101	1500	574	11	154	450	82
**Annual number of IVF cycles**	2012-2018	84064	33092	1430000	105000	100000	454893	··	41995	306197	9000

[a] From current reports in Table S5. Abbreviations: GDP: gross domestic product; IVF: *in vitro* fertilization.

Table S5. Infertility prevalence of the selected countries

	The first author's name	Research time	Methods	Population	Scale	Infertility prevalence
**By country**						
Australia	HP Collins	2003	National estimates	Men aged ≥ 40 years	··	8-9%
	Ashok Agarwal	2015	National estimates	Couples	··	15%
Canada	Balakrishnan TR	1984	Cross-sectional study	Women aged 18-44 years	··	5·40%
	Dulberg CS	1992	Cross-sectional study	Women aged 18-44 years	··	8·50%
	Tracey Bushnik	2009-2010	CCHS	Married and common-law couples with a female partner aged 18–44	4412 couples	11·5-15·7%
China	Jun Gao	2001	Cross-sectional study	Women aged 15-49 years	28511	17·13%
	Xiaoying Zheng	2001	Cross-sectional study	First married women	3414	18·00%
	Z Zhou	2010-2011	Cross-sectional study	Women aged 20-49 years	17275	15·50%
	Qinqin Meng	1980-2012	Meta-analysis	Newlyweds	27 studies	12·50%
	Jie Qiao	2011	National estimates	Women aged 15-45 years	··	15-20%
	Jie Qiao	2011	National estimates	Men aged 15-45 years	··	10-12%
	Qinqin Meng	2009-2012	Prospective study	Newlyweds	1627	13·60%
	National Health Commission of the People's Republic of China	2013	National estimates	Population of childbearing age	··	7-10%
	National Bureau of Statistics	2021	National estimates	Population of childbearing age	··	12-18%
France	P Thonneau	1991	Cross-sectional study	Women aged 15-45 years	867	14·10%
	R Slama	2007-2008	Multicentre survey	Women aged 18-44 years	1686 couples	24%
India	WHO	1980	National estimates	Population of childbearing age	··	3%
	WHO	1984	National estimates	Population of childbearing age	··	8%
	S Ganguly	1998-1999	Cross-sectional study	Women married for ≥ 5 years aged 20-49 years	1308	2·01%
	S Ganguly	2005-2006	Cross-sectional study	Women married for ≥ 5 years aged 20-49 years	1364	1·85%

	Paul C Adamson	2005-2006	Cross-sectional study	Women aged 15-30 years	897	12·60%
	Ashwini Katole	2013-2015	Cross-sectional study	Women in 15-49 years old	570	8·9% (2 years)
	Indian Society of Assisted Reproduction	2018	National estimates	Population of childbearing age	··	10-14%
Japan	Suzumori K	2001	National estimates	Population of childbearing age	··	10%
	Harumi K	2009	National estimates	Population of childbearing age	··	10-15%
Singapore	Seong Feei Loh	2013	National estimates	Population of childbearing age	··	14·29%
South Korea	Lee T H	2003	National estimates	Population of childbearing age	··	13·50%
	Weissman Ariel	2004	National estimates	Population of childbearing age	··	13%
	Insun Jang	2018	National estimates	Population of childbearing age	··	13·50%
the United States	E H Stephen	1982	Cross-sectional study	Women aged 15-44 years	··	8·40%
	E H Stephen	1988	Cross-sectional study	Women aged 15-44 years	··	8·40%
	E H Stephen	1995	Cross-sectional study	Women aged 15-44 years	10847	10·20%
	Anjani Chandra	2002	Cross-sectional study	Women aged 15-44 years	7643	7·40%
	National Center for Health Statistics	2015-2017	Cross-sectional study	Married women aged 15-49 years	··	8·80%
	Centers for Disease Control and Prevention	2019	National estimates	Women aged 15-44 years	··	10·00%
United Kingdom	David B Dunson	2004	Prospective study	Married women aged 19-26 years	782 couples	8%
	David B Dunson	2004	Prospective study	Married women aged 27-34 years	782 couples	13-14%
	David B Dunson	2004	Prospective study	Married women aged 35-39 years	782 couples	18%
	J Datta	2010-2012	Cross-sectional study	Women aged 16-74 years	8869	10·10%
	J Datta	2010-2012	Cross-sectional study	Men aged 16-74 years	6293	12·50%
	National institute for Health and Care Excellence	2013	National estimates	Population of childbearing age	··	14·29%

Table S6. Comparison of different patterns on infertility

	Male infertility			Female infertility		
	Mean[a]	95% CI		Mean[a]	95% CI	
		Lower	Upper		Lower	Upper
Policy orientation						
Economic support-oriented	-2·95	-11·05	5·16	-5·42	-14·04	3·20
Social security-oriented	2·20	-2·77	7·17	6·71	-1·50	14·92
Balanced	-0·57	-4·99	3·85	8·81	1·37	16·25
Implementation pace						
Gradual-promotion	-1·35	-2·85	0·14	4·33	-4·32	12·98
Simultaneous-promotion	4·84	2·26	7·43	3·28	-7·47	14·03

[a] Covariates appearing in the model are evaluated at the following values: Infertility baseline = 1.192000.

Abbreviation: CI: confidence interval.

Appendix 1. Data source of table S4

Total fertility rate

World Bank Database

https://data.worldbank.org.cn/indicator/SP.DYN.TFRT.IN?view=chart

Male first marriage age & Female first marriage age

the United States

Source: statista Database

https://www.statista.com/statistics/371933/median-age-of-us-americans-at-their-first-wedding/

Australia

Source: abs Database

https://www.abs.gov.au/statistics/people/people-and-communities/marriages-and-divorces-australia/latest-release

Canada

Source: Canada Cardus Database

https://www.cardus.ca/research/family/reports/the-canadian-marriage-map/

China

Source: World Bank Database

https://databank.worldbank.org/data/reports.aspx?source=312&series=SH.FPL.FMAR.Q1.ZS

France

Source: France Insee Database

https://www.insee.fr/en/statistiques/2382603?sommaire=2382613

India

Source: statista Database

https://www.statista.com/statistics/678453/mean-age-at-marriage-by-gender-and-region-india/

Japan

Source: japan data Database

https://www.nippon.com/en/japan-data/h00759/

South Korea

Source: statista Database

https://www.statista.com/statistics/1112935/south-korea-median-age-at-first-marriage-by-gender/

Singapore

Source: statista Database

https://www.statista.com/statistics/625108/median-age-of-first-time-marriages-in-singapore/

United Kingdom

Source: United Kingdom National Bureau of Statistics

https://www.ons.gov.uk/peoplepopulationandcommunity/birthsdeathsandmarriages/marriagecohabitationandcivilpartnerships/bulletins/marriagesinenglandandwalesprovisional/2016

First childbearing age

Australia

Source: government reports

https://www.aihw.gov.au/getmedia/7a8ad47e-8817-46d3-9757-44fe975969c4/aihw-per-97.pdf.aspx?inline=true

Canada

Source: statistics Canda Database

https://www150.statcan.gc.ca/n1/pub/91-209-x/2018001/article/54956-eng.htm

China

Source: Springer

https://link.springer.com/article/10.1007/s42379-019-00022-9/tables/1

France

Source: statista Database

https://www.statista.com/statistics/957266/mothers-age-at-childbirth-france/

India

Source: World Bank Database
https://databank.worldbank.org/reports.aspx?source=312&series=SH.FPL.FBRT.Q1.ZS#
Japan
Source: Mean Age of Mother, 1970–2000
https://www.cdc.gov/nchs/data/nvsr/nvsr51/nvsr51_01.pdf
South Korea
Source: statistics Korea Database
http://kostat.go.kr/portal/eng/pressReleases/8/10/index.board?bmode=read&aSeq=273490&pageNo=4&rowNum=10&amSeq=&sTarget=title&sTxt=
Singapore
Source: Article
chrome-extension://ikhdkkncnoglghljlkmcimlnlhkeamad/pdf-viewer/web/viewer.html?file=https%3A%2F%2Fwww.strategygroup.gov.sg%2Fimages%2FPress%2520Release%2520images%2FPDFs%2Fmarriage-and-parenthood-trends-in-singapore.pdf
United Kingdom
Source: United Kingdom National Bureau of Statistics
https://www.ons.gov.uk/peoplepopulationandcommunity/birthsdeathsandmarriages/livebirths/bulletins/birthcharacteristicsinenglandandwales/2017

National income level & Development Status
Source: World Bank Database
https://data.worldbank.org/indicator/NY.GNP.PCAP.CD

Per capita GDP ($)
Source: World Bank Database
https://data.worldbank.org.cn/indicator/NY.GDP.PCAP.CD?view=chart

Health expenditure (% of GDP)

Source: indexmundi Database
https://www.indexmundi.com/g/r.aspx?v=2225&l=zh

Per capita medical and health expenditure ($)
Source: knoema Database
https://cn.knoema.com/atlas/topics/%e5%81%a5%e5%ba%b7/%e5%8c%bb%e7%96%97%e4%bf%9d%e5%81%a5%e6%94%af%e5%87%ba/%e4%ba%ba%e5%9d%87%e5%8c%bb%e7%96%97%e6%94%af%e5%87%ba

Average cost of IVF ($)
Source: sunshine egg donating agency
https://www.eggdonors.asia/blog/how-much-ivf-cost/

Number of assisted reproductive institutions
Source: Nova IVF Fertility
https://www.novaivffertility.com/researchpdf/IFFS-Surveillance-2019.pdf

Annual number of IVF cycles
Source: Google Scholar, NCBI, National Centers for Disease Control and Prevention, Foresight Industry Research Institute, etc.
India https://palashivf.com/2017/02/21/ivf-treatment-market-india/
China https://bg.qianzhan.com/report/detail/459/190408-e9456aff.html
South Korea https://www.ncbi.nlm.nih.gov/pmc/articles/PMC5395552/
Japan https://onlinelibrary.wiley.com/doi/full/10.1002/rmb2.12358
France https://www.eshre.eu/-/media/sitecore-files/Press-room/ART-fact-sheet-2020-data-2016.pdf?la=en&hash=AB68A67B4FEA7723F2125B02BCB93FB837139CD4
United Kingdom
https://www.eshre.eu/-/media/sitecore-files/Press-room/ART-fact-sheet-2020-data-2016.pdf?la=en&hash=AB68A67B4FEA7723F2125B02BCB93FB837139CD4
the United States https://www.cdc.gov/art/artdata/index.html

Canada https://healthydebate.ca/2019/02/topic/funded-ivf-ontario

Australia https://newsroom.unsw.edu.au/news/health/almost-one-20-babies-australia-born-through-ivf

Appendix 2. Infertility-related policies in 10 selected countries (including classification and sources)

A. Australia

**Dimension**	**Index**	**Policies**	**Year**	**Grade**	**Source**
Economic Support	Insurance coverage	Semen analysis and clinical examination during pregnancy are partly reimbursed by the National Medical Insurance (MBS). The grouping in MBS is: P8-infertility and pregnancy test.	1992	II	http://www.mbsonline.gov.au/internet/mbsonline/publishing.nsf/Content/Home
		With the support of the National Medical Insurance (MBS) and Drug Benefits (PBS), all couples are eligible for partial compensation for unlimited ART treatments, regardless of the age of the parents or the number of previous treatment attempts or the current children's limit.	2000	II	Assisted reproductive technology: public funding and the voluntary shift to single embryo transfer in Australia
		In Australia, some of the costs of IVF services are covered by Medicare, as well as private health insurance policies2.	-	-	https://www.iselect.com.au/health-insurance/ivf/
		ART is introduced into the Extended Medical Insurance Safety Net (EMSN), which can reimburse 80% of out-of-hospital medical insurance out-of-pocket expenses.	2004	III	Assisted reproductive technology: public funding and the voluntary shift to single embryo transfer in Australia
	Financial assistance	-	-	-	-
	Economic reward	The Baby Bonus is paid to families following the birth or adoption of a baby. It is payable to: a parent of the baby; families who have care of a newborn child within 13 weeks of the child's birth and who are likely to have care of the child for no less than 13 weeks; and families who have a child entrusted to their care for adoption before the child is two years of age. Usually, the payment is made as a one-off lump sum payment of $4,133. For multiple births, the Baby Bonus is paid for each child; No income or assets tests apply.	2004	II	https://aifs.gov.au/publications/archived/954#925
		The baby bonus is $5,000, but only families with an annual income of less than $150,000 are eligible for the bonus.	2008	III	https://www.demographic-research.org/volumes/vol42/18/default.htm
		The baby bonus is US$3,000, and the family's annual income needs to be as low as US$75,000. At the same time, the baby fund can only be obtained if the mother cannot get paid parental leave.	2012	III	https://www.demographic-research.org/volumes/vol42/18/default.htm

	Infertility diagnosis and treatment leave security	Adding to these difficulties, leave for IVF treatment is not a legitimate use of personal leave under the Fair Work Act 2009 (Cth) (‘FW Act’), as women undergoing treatment are neither ‘ill’ nor ‘injured’.1	2009	I	http://www.unswlawjournal.unsw.edu.au/wp-content/uploads/2018/09/Hvala.pdf
Social security	Parental leave security	Australian women’s paid maternity leave for 18 weeks (minimum wage level)	2012	II	https://www.un.org/en/development/desa/population/events/pdf/expert/24/Policy_Briefs/PB_Australia.pdf
		A two-week paid maternity leave (minimum wage level) was implemented for the father or partner of a childbirth woman	2013	III	https://www.un.org/en/development/desa/population/events/pdf/expert/24/Policy_Briefs/PB_Australia.pdf
		All employees are entitled to 10 days of home care leave each year	-	-	https://www.un.org/en/development/desa/population/events/pdf/expert/24/Policy_Briefs/PB_Australia.pdf
		So-called “flextime” is also widely available in Australia. By working longer hours (without pay), workers can build up future leave credits. Parents often use flextime credits to cover the longer leave required during school holidays.	-	-	https://www.un.org/en/development/desa/population/events/pdf/expert/24/Policy_Briefs/PB_Australia.pdf
	Child care and education security	Child care tax rebate	2004	I	https://aifs.gov.au/publications/archived/954#925
		Those who started receiving Parenting Payment before July 2006 are eligible to continue to receive Parenting Payment until their youngest child turns 16 years (subject to continuing eligibility). Principal carers who went on to Parenting Payment (Partnered) on or after 1 July 2006 receive the Parenting Payment until their youngest child is 6 years old. When their youngest child turns 6 years, these parents need to test their eligibility for another income support payment if they still require such support. People on unemployment-related payments	2006	II	https://aifs.gov.au/publications/archived/954#925

		who are principal carers have an obligation to look for part-time work of at least 15 hours per week. Principal carers who are single parents and went on to the Parenting Payment (Single) on or after 1 July 2006 are eligible for Parenting Payment until their youngest child turns 8 years. At that time, they also need to test their eligibility for another income support payment, usually an unemployment-related payment (e.g., Newstart Allowance).			
	Life security	Family Tax Benefit (FTB). There are two parts to FTB:Part A and **Part B.** We pay Family Tax Benefit (FTB) Part A per child. The amount we pay you depends on your family's circumstances. If the baby is raised by the family before one year old or the family adopts children of any age, they can receive additional benefits called Newborn Upfront Payment and Newborn Supplement.<br>FTB B will provide additional assistance to single parents, non-parent dependents (including grandparents), and couples with only one source of income. Their claims are subject to an income test, and the amount received will depend on the age of the youngest child.	-	-	https://www.servicesaustralia.gov.au/individuals/services/centrelink/family-tax-benefit
		The National Initiative for Perinatal Depression has been developed to develop information and training materials for health professionals who will screen and treat new mothers and pregnant women with perinatal depression.	2008	II	https://www1.health.gov.au/internet/main/publishing.nsf/Content/mental-perinat
Service Package	Maternal and child health service	Maternal health care in Australia includes prenatal, mid- and post-natal care for women and babies up to six weeks after birth.<br>This care is provided in a variety of public and private environments, and is supported by a service capacity framework, labor, funding, information and data, and technical infrastructure.	2010	II	https://www1.health.gov.au/internet/main/publishing.nsf/Content/maternityservicesplan
		Children of all ages must receive the latest child immunizations, otherwise the family will not be able to receive Family Tax (FTB) Part A and childcare assistance.	2016	II	https://www.aph.gov.au/about_parliament/parliamentary_departments/parliamentary_library/pubs/rp/budgetreview201516/vaccination
	Assisted reproducti	The Victorian Department of Health and Human Services funded the establishment of the Victorian Assisted Reproductive Therapy Agency (VARTA)	-	-	https://www.varta.org.au/about-varta

Technology development guarantee	ve technology development	ART clinics must comply with the FAS standards established by the Australian Fertility Society (FAS) and the "Code of Ethics for the Use of Assisted Reproductive Technology in Clinical Practice and Research" established by the National Health and Medical Research Council (NHMRC)	-	-	https://www.varta.org.au/regulation/history-victorian-art-regulation
		Australia has legislated and regulated the implementation of ART technology. In 1984, the "Infertility (Medical Procedure) Act" was promulgated, the "Infertility Treatment Act" was promulgated in 1995, the "Assisted Reproductive Treatment Act 2008" was promulgated in 2008, and the "Infertility Treatment Act 2008" was promulgated in 2016. The 2008 Assisted Reproductive Law was revised	1984	II	https://www.varta.org.au/regulation/history-victorian-art-regulation
		The National Perinatal Epidemiology & Statistics Unit (NPESU) was established in 1979 and continues to be a leading source of statistical and epidemiological research in reproductive medicine, pregnancy, childbirth, and the health and care of newborns. It contains the Australian and New Zealand Assisted Reproductive Database (ANZARD)	1979	II	https://npesu.unsw.edu.au/about-us
Reproductive health promotion	Reproductive health education and protection	The National Health and Physical Education Curriculum was announced as part of the third phase of the National Curriculum Development Plan, and sex education was included.<br>In most states and regions, content related to sex education is mandatory before the age of 10.	2010	II	https://www.latrobe.edu.au/__data/assets/pdf_file/0019/148060/Sexual-Education-in-Australia-2011.pdf
		Strengthen strategies and action plans for addressing indigenous people's sexually transmitted diseases and territorial sexually transmitted infections	2014	II	https://ama.com.au/position-statement/sexual-and-reproductive-health-2014

B. Canada

Dimension	Index	Policies	Year	Grade	Source
		Quebec implements tax credits for infertility treatment costs	-	-	https://www.revenuquebec.ca/en/citizens/income-tax-return/completing-your-income-tax-return/completing-your-income-tax-return/line-by-line-help/451-to-480-refund-or-balance-due/line-462/point-11/
		Quebec became the first jurisdiction in Canada to provide comprehensive insurance for IVF.	2010	II	https://www.cadth.ca/sites/default/files/pdf/Public_Funding_IVF_es-14_e.pdf
		Manitoba offers a Fertility Treatment Tax Credit where 40% of treatment fees can be claimed to a maximum credit of $8,000.	2013	II	https://financialpost.com/personal-finance/does-your-province-cover-the-cost-of-infertility-treatments?r
Economic Support	Insurance coverage	While B.C. does not cover IVF, its Medical Services Plan does cover minor fertility treatments and some diagnostic tests.	2013	II	https://financialpost.com/personal-finance/does-your-province-cover-the-cost-of-infertility-treatments?r
		the Ontario will become only the second province in Canada to fund in-vitro fertilization for people unable to have children otherwise. The province will pay for the cost of one "cycle" of IVF treatment per patient suffering from "all forms of infertility".	2014	II	https://financialpost.com/personal-finance/does-your-province-cover-the-cost-of-infertility-treatments?r
		Ontario offers a program that provides funding to participating fertility clinics across the province to cover treatment costs for eligible residents. In-vitro fertilization (IVF): One treatment cycle per patient, which includes the one-at-a-time transfer of all viable embryos. Artificial insemination (AI): No limit on the number of treatment cycles. The plan does not cover the cost of fertility drugs, the cost of genetic testing, and the storage of sperm, eggs and embryos.	-	-	https://www.ontario.ca/page/get-fertility-treatments#section-2

	Financial assistance	-	-	-	-
	Economic reward	Québec has implemented the "Baby Bonus Package". Parents with a new child in the family receive cash from the government immediately after the child is born (or adopted).<br>Parents receive 500 Canadian dollars for the first or second childbirth.<br>For third or higher parities, pay 375 Canadian dollars in 8 quarters over 5 years, for a total of 3,000 Canadian dollars.<br>From May 1, 1989, on the first birthday of the second child, the second remittance amounted to 500 Canadian dollars.<br>The final amount gradually increased until it reached 8000 Canadian dollars in 1992 (400 Canadian dollars paid in 21 quarters).	1988	II	A 'Natural Experiment' on the Economics of Storks: Evidence on the Impact of Differential Family Policy on Fertility Rates in Canada
		"Canadian Child Benefit" is a family policy related to raising children administered by the Canada Revenue Agency.<br>All parents with children under 18 can apply.<br>This benefit is tax-free and the state pays it monthly.<br>The amount of welfare is related to the number of children, place of residence, net family income, and other welfare status enjoyed by the children.<br>Children with high parity (such as three births and above) can receive additional subsidies.	2013	III	Analysis of Foreign Family Policies Affecting Fertility
Social security	Infertility diagnosis and treatment leave security	-	-	-	-

Parental leave security	Maternity leave varies from province to province, but it is usually 15 or 18 weeks. Canada's maternity leave allowance is paid by federal unemployment insurance. Court precedents guarantee that employers should provide reasonable assistance measures to women workers during pregnancy.	1971	II	Canadian Women Workers' Responsibility Policy
	Women workers shall continue to enjoy the relevant benefits during maternity leave on an equal footing, and when returning to positions of the same value after taking maternity leave, they shall consider the remuneration and benefits of their labor status.	1992	II	Canadian Women Workers' Responsibility Policy
	Canadian provinces stipulate that the parental leave can be applied for by both the father and the mother after the child is born about one year or so. Parental leave belongs to the nature of leave without pay. The government pays a childcare allowance.	-	-	Canadian Women Workers' Responsibility Policy
	In Quebec, there are 52 weeks of parental leave after the parents are combined, and the parental leave belongs to the father, that is, the father has another 5 weeks of parental leave.	-	-	Canadian Women Workers' Responsibility Policy
	Quebec's Labor Standards Law regulates maternity leave and parental leave. There is also a short-term vacation of 5 days within 15 days after childbirth or adoption. The requirement is that the employed worker must have 60 days of uninterrupted seniority. Among the five days of vacation, the first two days are paid vacations for the needs of laborers to give birth or adopt children.	-	-	Canadian Women Workers' Responsibility Policy
	British Columbia stipulates that there is a five-day family care leave for full-time job-worship, including child care, health and education. Workers can thus increase the period of childcare. Prince Edward Island and New Brunswick provide three days of family care leave in a year, which can also increase parental leave.	-	-	Canadian Women Workers' Responsibility Policy

	After abolishing the universal family allowance system, Quebec adopted a compensation measure—increasing childcare services and bearing the cost of daycare, that is, regardless of family income, a childcare policy of $5 per child per day.	1998	II	A 'Natural Experiment' on the Economics of Storks: Evidence on the Impact of Differential Family Policy on Fertility Rates in Canada
Child care and education security	Facing the shortage of childcare facilities, the Canadian Federal Government coordinated with governments at all levels to build more childcare centers. In 2005, it proposed to establish 125,000 childcare service centers within five years from 2006.<br>Since 2007, 25,000 spaces have to be generated each year.<br>It also provides a way to attract companies to set up in-company childcare facilities with a tax exemption of 10,000 yuan, in order to obtain a response from the business community.	2005	II	Canadian Women Workers' Responsibility Policy
	Canada implements a national comprehensive childcare subsidy. Parents can choose to receive child care allowance or send their children to public childcare centers, regardless of the parents' income.<br>The childcare subsidy pays 100 Canadian dollars a month for each child until the age of six.<br>The childcare subsidy can be used to pay for the cost of childcare institutions, and can also be used to purchase learning materials for young children.<br>If the parents bring their children at home, they can also be deposited into the children's education savings fund.	2006	III	Canadian Women Workers' Responsibility Policy
Life security	The tax reduction policy for children under the age of 16 is reintroduced. The tax reduction for the first child is 1,830 Canadian dollars, and the tax reduction for each subsequent child is 1,370 Canadian dollars.	1986	II	A 'Natural Experiment' on the Economics of Storks: Evidence on the Impact of Differential Family Policy on Fertility Rates in Canada

		Introduced family tax exemption allowance for newborns. When the first or second child is born (or adopted), each family receives 500 Canadian dollars. For the third or more births, each family will receive 8 quarterly payments of 375 Canadian dollars (3000 Canadian dollars).	1988-1998	II	A 'Natural Experiment' on the Economics of Storks: Evidence on the Impact of Differential Family Policy on Fertility Rates in Canada
		Families with children under the age of 18 can now enjoy a family tax reduction. The maximum deduction is $965, and the maximum deduction is 4% for families with incomes higher than $26,000.	1988-1998	II	A 'Natural Experiment' on the Economics of Storks: Evidence on the Impact of Differential Family Policy on Fertility Rates in Canada
		Families with children under the age of 18 can now enjoy a family tax reduction. The maximum deduction is $965, and the maximum deduction is 4% for families with incomes higher than $26,000.	2010	III	https://financialpost.com/personal-finance/does-your-province-cover-the-cost-of-infertility-treatments?r
		Families caring for a child with a physical or mental disability under the age of 18 can also receive an additional tax-free "disabled child allowance".	2013	III	Analysis of Foreign Family Policies Affecting Fertility
Service Package	Maternal and child health service	According to the Canadian Health Act, Canada's health care is provided through the provincial and territorial publicly funded health care system, which includes maternal and child health services.	1984	II	https://web.archive.org/web/20031205153216/http://laws.justice.gc.ca/en/C-6/
Technology development guarantee	Assisted reproductive technology development	-	-	-	-
Reproductive health promotion	Reproductive health education	The Public Health Agency of Canada has established the Division of Sexual Health and Sexually Transmitted Infections, which works with provinces, non-governmental organizations and healthcare providers to improve and maintain the sexual health and	2006	II	https://www.canada.ca/en/health-canada/services/health-concerns/diseases-conditions/sexually-transmitted-infections.html

and protection	well-being of Canadians by helping prevent and prevent AIDS, and control sex. Spread infection and its complications.			
	The Governor of Ontario, Wayne, promulgated the "Ontario Health and Physical Education Curriculum Standards for Grades One to Eight (2015 Edition)" and began to implement in September of the same year. At the same time, sex education was added to the health and physical education curriculum.	2015	II	A Review of Elementary School Sex Education in Ontario, Canada

C. China

Dimension	Index	Policies	Year	Grade	Source
Economic Support	Insurance coverage	The diagnosis and treatment of infertility and sexual dysfunction were not covered by basic medical insurance.	1999	I	http://www.nhsa.gov.cn/art/2020/10/10/art_26_3698.html
		Some provinces provided subsidies for parents who lost their only child and required for assisted reproduction procedure.	2020	I	http://www.nhsa.gov.cn/art/2020/10/10/art_26_3698.html
		Drugs that enhance sexual function were not included in Pharmaceutical Benefits Scheme.	2020	I	http://www.nhsa.gov.cn/art/2020/7/31/art_37_3387.html
	Financial assistance	Population requiring for assisted reproduction procedure were provided with guidance and financial support.	2013	II	http://www.nhc.gov.cn/rkjcyjtfzs/zcwj2/201312/2a7b17d627354404922d8689a56c879c.shtml
	Economic reward	Puerpera nutrition allowance were provided for working women and the setting of the allowance was based on average income of local employees.	2011	II	http://www.gov.cn/flfg/2010-10/28/content_1732964.htm
Social security	Infertility diagnosis and treatment leave security	Leave for infertility treatment was not included in leave covered by birth insurance.	2011	I	http://www.gov.cn/flfg/2010-10/28/content_1732964.htm
	Parental leave security	Female workers enjoyed the maternity allowance during maternity leave lawfully.	2011	II	http://www.gov.cn/flfg/2010-10/28/content_1732964.htm
		The allowance in paternity leaves covered only a few provinces.	2011	II	http://www.gov.cn/flfg/2010-10/28/content_1732964.htm
		According to the law, employers could not reduce wages of female workers or dismiss her because of her pregnancy and breast-feeding.	2012	II	http://www.gov.cn/zhengce/2020-12/27/content_5574753.htm

	Child care and education security	The pilot reforms of quality education and educational equality were carried out.	2010	II	http://www.gov.cn/jrzg/2010-07/29/content_1667143.htm
		To reduce the burden of parenting family, employment security, tax reduction and housing subsidies were put into effect.	2016	II	http://www.gov.cn/zhengce/content/2017-01/25/content_5163309.htm
	Life security	The system of subsistence allowances for urban and rural residents were established.	2007	II	Family Support Policy Reform and Perfection after the Implementation of Two 一 child Policy
		Economic and social supports were provided for zero-employment family.	2006	II	Family Support Policy Reform and Perfection after the Implementation of Two 一 child Policy
		The system of "Five Guarantees" were implemented in rural areas.	2006	II	Family Support Policy Reform and Perfection after the Implementation of Two 一 child Policy
Service Package	Maternal and child health service	Law of the Peoples Republic of China on Maternal and Infant Health Care was enforced.	1995	II	http://www.npc.gov.cn/zgrdw/npc/xinwen/2017-11/28/content_2032724.htm
		The Law of the People's Republic of China on Maternal and Infant Health care was amended for the first time.	2009	II	http://www.npc.gov.cn/zgrdw/npc/xinwen/2017-11/28/content_2032724.htm
		The Law of the People's Republic of China on Maternal and Infant Health care was amended for the second time.	2017	III	http://www.npc.gov.cn/zgrdw/npc/xinwen/2017-11/28/content_2032724.htm
Technology development guarantee	Assisted reproductive technology	Artificial insemination could not be carried out in any health care institution except for scientific research.	1989	I	http://www.law-lib.com/law/law_view1.asp?id=50494
		Assisted reproductive technology were allowed in China, and strict examination and approval were required to obtain professional qualifications. The qualifications should be approved by National Health and Family Planning Commission of the People's Republic of China.	2001	I	http://www.nhc.gov.cn/yzygj/s3576/200804/ca4f5f1f7a224cadb045e39b200fa519.shtml

	development	Audit and manage procedures of ART and sperm bank were improved.	2003	Ⅰ	http://www.nhc.gov.cn/bgt/pw10303/200708/6998a89f66c247469a179482dd9f930c.shtml
		The qualifications of ART could be approved by provincial health development planning commission.	2007	II	http://www.nhc.gov.cn/bgt/pw10709/200709/857e40d3228447199d2ddc0d3b6639ae.shtml
		The Ministry of Science and Technology issued "Development and Reproductive Research During the 12th Five-Year Plan Period" which emphasisd the safety of assisted reproductive technology.	2012	II	http://www.most.gov.cn/kjbgz/201207/t20120716_95627.html
		Prevention and treatment of infertility were included in national key scientific research project.	2018	II	http://www.most.gov.cn/tztg/201801/t20180119_137801.html
Reproductive health protection	Reproductive health education and protection	Sexual and reproductive health education was included in compulsory curriculum.	2011	II	http://www.gov.cn/jrzg/2011-08/09/content_1921982.htm
		Sex education for children and adolescents were highlighted to reduce prevalence of sexually transmitted diseases.	2017	III	http://www.gov.cn/zhengce/2017-04/13/content_5185555.htm#1

D. France

Dimension	Index	Policies	Year	Grade	Source
Economic Support	Insurance coverage	The French healthcare system covers medically assisted reproduction (AMP) of women before 43, which can enable infertile couples to give birth to a child, including up to 6 cycles of artificial insemination and 4 cycles of in vitro fertilization (IVF).	-	-	https://www.service-public.fr/particuliers/vosdroits/F31462
	Financial assistance	-	-	-	-
	Economic reward	Families in France who give birth to their first child can receive a monthly subsidy of about 177 euros. If they give birth to a second child within three years, the amount will rise to about 600 euros. The subsidy will continue until the child reaches 6 years old. If another child is born,<br>, The subsidy will increase to more than 900 euros and continue to be paid until the child reaches 18 years of age.<br>In France, the subsidy amount for the fourth child is the highest, after which the subsidy amount will decrease as the number of children increases.	1939	II	http://theory.people.com.cn/n/2012/1008/c136457-19190735.html.
Social security	Infertility diagnosis and treatment leave security	-	-	-	-
	Parental leave security	Family policy in France was institutionalized in 1938 with the introduction of the Family Code.	1938	II	http://www.ipss.go.jp/webj-ad/WebJournal.files/population/2003_6/9.Letablier.pdf
		Paternity leave: Since January 2002, fathers have been eligible for two weeks' paid leave following the birth of a child5<br>. Leave is paid for by social	2002	III	http://www.ipss.go.jp/webj-ad/WebJournal.files/population/2003_6/9.Letablier.pdf

	security as a replacement wage6 .			
	Parents of young children have the right to take relatively long parental leave. Parents with at least two children can take up to 36 months of parental leave. They can receive a fixed home allowance, which is slightly less than half of the minimum wage.	2004	III	https://www.ined.fr/en/everything_about_population/demographic-facts-sheets/focus-on/family-policy-france/
	The measures introduced in 2013 were in full swing with the reform of family allowances, and for families with a monthly income of more than 6,000 euros (US$6,814), this proportion is declining. For mothers with at least two children, the stay-at-home allowance paid during parental leave is now limited to 24 months instead of the previous 36 months, but the allowance can be extended if the father is on leave	2013	III	https://www.ined.fr/en/everything_about_population/demographic-facts-sheets/focus-on/family-policy-france/
	Parental leave: After maternity leave or adoption, parents have the right to take parental leave, or to work part-time (not less than sixteen hours per week) if they have been working for the same employer for at least one year.	-	-	http://www.ipss.go.jp/webj-ad/WebJournal.files/population/2003_6/9.Letablier.pdf
	Every employee has the right to take unpaid leave to care for his/her sick child under the age of sixteen. Legally, periods of leave may not exceed three days (or five days in specific cases), but this is a minimum. In the public sector, employees are allowed to take up to fourteen days a year off to care for a sick child.	-	-	http://www.ipss.go.jp/webj-ad/WebJournal.files/population/2003_6/9.Letablier.pdf
	The maternity leave for 1 child is 16 weeks, for the second child is 26 weeks; for twins is 34 weeks, and for 3 babies is 46 weeks.	-	-	http://www.ipss.go.jp/webj-ad/WebJournal.files/population/2003_6/9.Letablier.pdf
Child care and education security	Childcare allowances: A parental leave allowance (APE) is paid to compensate for loss of income during parental leave. Two other allowances, the AGED (allocation de garde d’enfant à domicile) and AFEAMA (aide aux familles pour l’emploi d’une assistante maternelle agréée) were also introduced in the	1980	II	http://www.ipss.go.jp/webj-ad/WebJournal.files/population/2003_6/9.Letablier.pdf

	1980s to alleviate the cost of childcare when parents employ a childminder or a nanny in their home.			
	the government decided to increase childcare allowances and to give tax concessions to help families meet the costs of individualised childcare arrangements, such as registered childminders or nannies in the child’s home.	1994	III	http://www.ipss.go.jp/webj-ad/WebJournal.files/population/2003_6/9.Letablier.pdf
	the Ministry of Family Affairs decided to increase substantially the number of places in crèches: a budget of 228 million euros was devoted to public childcare facilities. Nursery schools are free of charge, but parents have to Activities subsidised by this funding are also provided for older children after school hours, on Wednesdays, and during holidays. pay for lunches and for care out of school hours which is usually organised by local authorities and subsidised by family policy funding.	2001	III	http://www.ipss.go.jp/webj-ad/WebJournal.files/population/2003_6/9.Letablier.pdf
	Child care services provide free public schools for all children aged 3 to 6 in pre-school education	-	-	http://www.ipss.go.jp/webj-ad/WebJournal.files/population/2003_6/9.Letablier.pdf
	In France, the child’s childcare fees are reimbursed by the government. If it is twins or multiples, the government will also pay to hire a nanny to take care of them at home.	-	-	http://www.ipss.go.jp/webj-ad/WebJournal.files/population/2003_6/9.Letablier.pdf
	Tax deductions: In France, childcare expenses for children under 7 can be deducted from the tax base payable, and the cost of childcare institutions built by enterprises can be deducted before tax.	-	-	http://www.ipss.go.jp/webj-ad/WebJournal.files/population/2003_6/9.Letablier.pdf
Life security	Increase the number of jobs available to mothers	1970	II	http://www.ipss.go.jp/webj-ad/WebJournal.files/population/2003_6/9.Letablier.pdf

Two public transfer payments are aimed at supporting the neediest families: the RMI (Revenu minimum d'insertion) and the API (Allocation de parent isolé). The RMI is a minimum income, a welfare supplement paid to lower-income families. RMI recipients are required to follow a training scheme or to take a job if offered one. The API is a lone-parent allowance introduced in 1976 to guarantee lone parents a minimum monthly income. The API is a means-tested allowance paid for one year, and is renewable until the child is three years old.	1976	II	http://www.ipss.go.jp/webj-ad/WebJournal.files/population/2003_6/9.Letablier.pdf
In addition to the normal family allowances, special allowances are designed to compensate disabled children and children from single-parent families. Allowances are also given to families every year to compensate for expenses related to the new school year.	1997	III	http://www.ipss.go.jp/webj-ad/WebJournal.files/population/2003_6/9.Letablier.pdf
Family allowances: They are paid to families to partially compensate for the cost of having children. They are paid to families with at least two children, up to age of 16 (the limit of compulsory schooling). They can be paid for children aged from 16 to 20 when they continue to attend school or university or are on training schemes.	1997	III	http://www.ipss.go.jp/webj-ad/WebJournal.files/population/2003_6/9.Letablier.pdf
Free Choice Compensation (CLCA) is a family subsidy that can be used by parents of two or more children. It can be paid to parents who choose to stop working or work less for up to three years. In 2004, the right to enjoy this benefit was extended to parents of one child, but the period was only six months	2004	III	https://www.ined.fr/en/everything_about_population/demographic-facts-sheets/focus-on/family-policy-france/
Tax Schemes for Expenditure on Children: Both schemes allow a progressive reduction in taxable income according to a unit scheme that allocates units according to family size and composition.	-	-	http://www.ipss.go.jp/webj-ad/WebJournal.files/population/2003_6/9.Letablier.pdf

Service Package	Maternal and child health service	-	-	-	-
Technology development guarantee	Assisted reproductive technology development	The draft law on bioethics provides for the extension of LDC to all women.	2019	II	https://www.service-public.fr/particuliers/vosdroits/F31462
Reproductive health promotion	Reproductive health education and protection	France will pay for condoms used to prevent sexually transmitted diseases. Schools are expected to provide 30 to 40 hours of sex education, and pass out condoms, to students in grades 8 and 9 (aged 15–16).	1973	II	https://en.wikipedia.org/wiki/Sex_education
		the French national health authority announce that it will partially reimburse the cost of male condoms obtained through prescription from a doctor or midwife. The aim is to combat the spread of HIV and other sexually transmitted diseases.	2018	II	https://www.commonwealthfund.org/publications/newsletter-article/2019/apr/france-cover-cost-condoms-combat-sexually-transmitted

E. India

Dimension	Index	Policies	Year	Grade	Source
Economic Support	Insurance coverage	Assisted reproductive technology (ART) was not covered by health insurance, and the president of the Assisted Reproduction Society in India said he wanted ART to be covered by health insurance.	2017	I	https://www.isarindia.net/
	Financial assistance	A government-run health insurance was implemented for poor citizen, called RSBY. Each family below the poverty line paid a registration fee of 30 rupees (42 cents) for biometric smart cards, and this family could get up to 30000 rupees for medical service fee.	2008	II	http://www.rsby.gov.in/overview.aspx
	Economic reward	The National Social Assistance Programme (NSAP) was implemented which included the National Maternity Allowance.	1995	II	https://nsap.nic.in/
		The Indira Gandhi Matritva Sahyog Yojana (IGMSY) programme was launched by the Ministry of Women and Child Development.The program is available to pregnant women 19 years of age or older, and they could receive 6000 rupees.	2010	II	http://niti.gov.in/writereaddata/files/document_publication/IGMSY_FinalReport.pdf
Social security	Infertility diagnosis and treatment leave security	-	-	-	-
	Parental leave security	Female employees have paid maternity leave for 12 weeks.	1961	II	https://labour.gov.in/sites/default/files/TheMaternityBenefitAct1961.pdf
		The Maternity Benefit (Amendment) Act, 2017 increased the duration of paid maternity leave available to working mothers from 12 weeks to 26 weeks.	2017	II	https://www.mondaq.com/india/Employment-and-HR/668308/Creche-Facility-Under-Maternity-Benefit-Amendment-Act-2017
		The Maternity Benefit Amendment Act makes it mandatory for employers to educate women about the maternity benefits available to them at the time of their appointment.	2017	II	https://labour.gov.in/whatsnew/maternity-benefit-amendment-act2017

	Child care and education security	The Maternity Benefit Amendment Act makes creche facility mandatory for every establishment employing 50 or more employees. Women employees would be permitted to visit the crèche 4 times during the day (including rest intervals)	2017	II	https://www.mondaq.com/india/Employment-and-HR/668308/Creche-Facility-Under-Maternity-Benefit-Amendment-Act-2017
		The Social Security Division, Ministry of Labour and Employment, has issued a circular dated November 17, 2018, wherein State governments have been requested to frame and notify Rules prescribing amenities and facilities required to be provided in a creche.	2018	II	https://www.mondaq.com/india/Employment-and-HR/668308/Creche-Facility-Under-Maternity-Benefit-Amendment-Act-2017
		The system of compulsory education was implemented, which covered children under 14 years old.	-	-	https://www.india.gov.in/my-government/constitution-india/constitution-india-full-text
	Life security	The National Social Assistance Programme (NSAP) was a welfare programme being administered by the Ministry of Rural Development. This programme was implemented in rural areas as well as urban areas.	1995	II	https://nsap.nic.in/circular.do?method=aboutus
Service Package	Maternal and child health service	In the slums of Banganor, there were mobile medical stations (mobiles) consisting of one doctor, two medical assistants and two nurses. A variety of basic medical services were provided for the residents, including prenatal examination, examination of women's diseases, contraceptive guidance and health education.	1999	I	Report on Reproductive Health in India
Technology development guarantee	Assisted reproductive technology development	In order to develop young doctors' skills and expertise in assisted reproduction, the embryologist and gynecologist Certification training Scholarships were set up.	2017	II	Expanding IVF treatment in India------need of the day!!
		The pesident of Indian Society for Assisted Reproduction appeal to develop and adopt innovative domestic “Made in India” treatments which could reduce the cost of treatment.	2017	II	Expanding IVF treatment in India------need of the day!!
Reproductive health protection	Reproductive health education and protection	-	-	-	-

F. Japan

Dimension	Index	Policies	Year	Grade	Source
	Insurance coverage	The South Korean government issued STD guidelines for South Korea in 2011 (updated in 2016)	-	-	https://www.oakclinic-group.com/english/funin_eg/fees_eg.html
		The National Health Insurance did not cover ART.	2004	I	https://www.eiu.com/graphics/marketing/pdf/Fertility-in-Japan-EIU.pdf
Economic Support	Financial assistance	Economic support for infertile women	1994	II	Jiang Juan, Comparative Study and Enlightenment on Legal Systems of Maternal and Child Health Care between China and Japan
		In 2004, couples earning less than 7.3 million yen (about $60,000) a year were eligible for 150,000 yen per treatment cycle, with no age limit and up to 100,000 yen per in vitro trial over five years for up to 10 trials.	2004	II	http://apps.who.int/iris/bitstream/10665/259941/1/9789290226260-eng.pdf?ua=1
		In 2016 and after, the number of years and times of receiving assisted reproductive technology within one year has not be restricted, and a maximum of 6 times of treatment has been subsidized; For those aged 40-42, a maximum of three treatments were subsidized.	2016	III	International policies related to assisted reproductive technology
		Okinawa subsidizes part of the cost of certain infertility treatments (in vitro fertilization and microinsemination) that were not covered	-	-	http://yabiku-ladys-clinic.jp/cli_fee/
		Japan's Health, Labor and Welfare Ministry said Friday it would seek more money from the government in its budget request for fiscal 2021 to increase subsidies for couples receiving infertility treatment.	2020	III	https://www.nippon.com/en/news/yjj2020092500413/?cx_recs_click=true
		The Japanese government would significantly expand existing subsidies for fertility treatment until public insurance be available, Prime Minister Yoshihide Suga alledged in Thursday.	2020	III	https://www.nippon.com/en/news/yjj2020101500819/?cx_recs_click=true
	Economic reward	When insured persons under the National Health Insurance (NHI) give birth, they will receive a childbirth benefit of 420,000 yen per child.	-	-	https://www.city.minato.tokyo.jp/easyjp/

					en/moving/nationalhealthinsurance/benefits.html
	Infertility diagnosis and treatment leave security	The company shall grant leave to employees who request it for infertility treatment.	2017	II	https://www.mhlw.go.jp/bunya/koyoukintou/pamphlet/dl/30k.pdf
Social security		Japanese women are entitled to six weeks of maternity leave before birth and eight weeks after, and receive the same benefits during maternity leave as they did before. Japanese women are entitled to 10 months of parental leave between the end of maternity leave and the child's first birthday.	1991	II	Legal System Construction of Maternity Leave in China
		Japanese men are legally entitled to eight weeks of parental leave, and the law encourages them to take it. If a man takes it while a woman is on maternity leave, he is entitled to another eight weeks of parental leave before the child is 14 months old.	-	-	Legal System Construction of Maternity Leave in China
	Parental leave security	In order to guarantee the right of all parents to parental leave for one year, Japan established the parental leave allowance.	1995	III	Legal System Construction of Maternity Leave in China
		The child-care leave system and promote the popularization of child-care leave were improved. For instance, if a child cannot attend a nursery, the child-care leave can be extended up to the age of two	2007	III	The Process of Japan's Childlessness and Its Policy Response
		Relevant laws and regulations have been revised and improved to prohibit enterprises from unfairly treating or discriminating against employees who take leave due to pregnancy or childbirth	2007	III	The Process of Japan's Childlessness and Its Policy Response
		Assistance programs for small and medium-sized enterprises were developed, and companies that actively devote their employees to parental leave and post-natal recovery were subsidized.	2007	III	The Process of Japan's Childlessness and Its Policy Response

	According to the diversity of people's life style, the establishment of "short-term formal employee system", flexible working system, remote office system and other systems provided a variety of work mode choice.	2007	III	The Process of Japan's Childlessness and Its Policy Response
	Establish a "subsidy for promoting women's activism" system to subsidize local governments that develop and implement plans to promote women's activism	2007	III	The Process of Japan's Childlessness and Its Policy Response
	The supervision and guidance of enterprises were strengthened so as to support women to return to work after childbirth; Opportunities were given for women who leave their jobs for childcare and other reasons to study again, creating conditions for them to enter the society again.	2007	III	The Process of Japan's Childlessness and Its Policy Response
	In Japan, employees are allowed five to 10 days a year (five days for one child and 10 days for two children) to take care of sick children. Before the reform, they took one day off, but after the reform, they can be on leave by half a day.	2016	III	A Rediscussion on Japan's Response to low Birth Rate Policy
Child care and education security	For families with many children, admission to nursery schools is free and priority is given after the third child.	2015	II	Outline of Countermeasures for a Society with fewer Children
	A new childcare system has been established to divide childcare service providers into "facility type" and "community type", and providing financial subsidies based on uniform standards and the same measures.	2015	II	The Process of Japan's Childlessness and Its Policy Response
	Single-parent families with an annual income of less than 3.6 million yen will be exempted from childcare fees for their second and subsequent children and will receive up to 10,000 yen a month in childcare subsidies	2016	II	A Rediscussion on Japan's Response to low Birth Rate Policy
	The plan is to increase the number of small child-care workers from 19 to 22, and improve the working environment for nursery governess.	2016	II	A Rediscussion on Japan's Response to low Birth Rate Policy
	To "support the business of combining work and child care" (enterprise-oriented child care business), enterprises are encouraged to establish and operate childcare facilities and provide infant care services for their employees with financial assistance from the government.	2016	II	The Process of Japan's Childlessness and Its Policy Response

		Regardless of the family income, education fees are free for children aged 3 to 5 who are enrolled in government-approved kindergartens and children aged 0 to 2 from low-income families who are exempt from resident tax. Children enrolled in non-designated kindergartens can receive up to 37,000 yen (three to five years old) and 42,000 yen (zero to two years old) per month as long as their parents can prove that they are working. Children attending private kindergartens receive 25,700 yen a month.	2019	III	A Rediscussion on Japan's Response to low Birth Rate Policy
		The Child Allowance is a means-tested in-cash transfer to households with children aged 6 years or younger. Established in 1972, the Child Allowance initially covered the third child and subsequent children below 18 years of age.	1972	II	http://www.ipss.go.jp/webj-ad/WebJournal.files/SocialSecurity/2002/02AUG/abe.pdf
	Life security	The family of the third generation is given a subsidy and income tax deduction for the renovation of the house.	2016	II	A Rediscussion on Japan's Response to low Birth Rate Policy
		Child allowance system: Children from age 0 to the age of graduation from junior high school in Japan (up to the first March 31 after the age of 15) are given different levels of subsidies	-	-	The Process of Japan's Childlessness and Its Policy Response
Service Package	Maternal and child health service	Japan has sound laws related to maternal and child health care, including: "Regional health care", "mother and child welfare law the act of preventing child abuse the child benefit act physically and mentally disabled policy basic law" infectious diseases prevention law "" school hygiene" education basic law "labor law" the act of raising a vacation, etc., basic covers the women's and children's psychological, physiological, social adaptation, such as nutrition, rehabilitation, all health problems.	1947	II	Comparative Study and Enlightenment on Legal Systems of Maternal and Child Health Care between China and Japan
		After pregnancy, women go to the health centers (centers) in their jurisdiction to receive free maternal and child Health manuals and child-rearing books, regularly attend free training courses for pregnant women, receive 14 free antenatal examinations, and have special personnel to keep in touch with pregnant women to answer their physical, mental and other health problems.	-	-	Current Situation and Enlightenment of Maternal and Child Health Care Service in Japan

		Children before the age of 3 receive free vaccinations against polio, measles, rubella, je, BCG and diphtheria at health institutions on a regular basis.	-	-	Current Situation and Enlightenment of Maternal and Child Health Care Service in Japan
Technology development guarantee	Assisted reproductive technology development	-	-	-	-
Reproductive health promotion	Reproductive health education and protection	Purity education stage: In 1946, illegal prostitution was banned and its prevention and protection measures; The 1947 Circular on the Implementation of Purity Education; Basic Essentials of Purity Education (1949); The Way purity Education is conducted, 1955	1946	I	Sex Education in Primary and Secondary Schools in Japan
		Establishment stage of sex education: guidebook of Sex Education in 1974; 1987 Circular on the Popularization of AIDS Knowledge	1974	II	Sex Education in Primary and Secondary Schools in Japan
		Comprehensive deepening of sex education: In 1992, primary school health textbooks were used for the first time since the Meiji era in Japan	1992	III	Sex Education in Primary and Secondary Schools in Japan

G. Singapore

Dimension	Index	Policies	Year	Grade	Source
Economic Support	Insurance coverage	Singapore has included assisted reproduction therapy in MediSave. MediSave is a national health savings plan those transfers 8 to 15 percent of a person's monthly income to a health savings account. For low-income workers, the government provides health savings to cover medical expenses for themselves or their families (parents, spouse and children). Whether assisted reproductive treatment is received on an outpatient basis or in hospital, the out-of-pocket portion of the costs may be paid using the MediSave account of the patient or spouse, with the following limits: S $6,000 (about RMB 29,000) for the first cycle, S $5,000 (about RMB 24,200) for the second cycle, s $4,000 (about RMB 19,300) for the third and subsequent cycles, In total, patients can withdraw up to S $15,000 from their or their spouse's MediSave account for assisted reproduction treatment.	1987	II	https://www.moh.gov.sg/cost-financing/healthcare-schemes-subsidies/medisave
		The Singapore Government has launched the ART Co-funding Scheme, which aims to provide funding to couples undergoing assisted reproduction treatment at public assisted reproduction centres, with one of the recipients having to be a Singaporean citizen. For couples who are both Singaporean citizens, the subsidy is 50 per cent of the cost of treatment up to three cycles, with a cap of S $3,000 per cycle. If one partner is a Singaporean citizen, the couple will receive 35 per cent of the cost of treatment.	2008	II	https://www.moh.gov.sg/news-highlights/details/response-to-adjournment-motion-on-providing-more-help-for-those-undergoing-ivf
		The Singapore government has further increased funding for the ART co-funding program, increasing funding for assisted reproduction therapy to 75 percent of the cost of treatment for couples who are both Singaporean citizens, with a cap of S $6,300 per cycle. For couples where one partner is a Singaporean citizen, funding is capped at 3,000 Singapore dollars per cycle of assisted reproductive treatment.	2013	III	https://www.pacificprime.sg/blog/in-vitro-fertilization-in-singapore/
		The Singapore government has introduced three cycles of egg freezing funding at 75 per cent of the total cost, capped at S $1,200 per cycle. For foreigners married to Singaporean citizens, the subsidy is capped at S $600 per egg freezing cycle.	2013	III	-

	The Singapore government has increased funding for assisted reproduction treatment and egg freezing: for assisted reproduction treatment, funding has been capped at S $7,700 per cycle; For egg freezing, the maximum funding is raised to S $2,200 per cycle.	2018	III	https://www.moh.gov.sg/news-highlights/details/response-to-adjournment-motion-on-providing-more-help-for-those-undergoing-ivf
	The government has lifted the age limit of 45 for artificial insemination and the number of times women over 45 can have access to assisted reproductive technology. For women who try assisted reproduction treatment or intrauterine insemination (IUI) before age 40, two of the six cycles of subsidized treatment can be performed after age 40. At the same time, the government is funding three cycles of intrauterine insemination (IUI) up to S $1,000 per cycle.	2020	III	https://www.moh.gov.sg/cost-financing/healthcare-schemes-subsidies/marriage-and-parenthood-schemes
Financial assistance	-	-	-	-
	Subsidies are offered to families having second and third children	2000	II	Implications of The International Population Policy Shift for China
	Subsidies are offered to families having their first to fourth child	2004	II	Implications of The International Population Policy Shift for China
Economic reward	The government pays a baby bonus. For children born before 2015, each child receives 6,000SGD $for the first and second children, and 8,000SGD $for the third and fourth children. No baby bonus is given to the fifth and subsequent children. For children born after January 1, 2015, 8000SGD$ is paid to each child for the 1st ~ 2nd children, 10000SGD$for the 3rd ~ 4th children and 10000SGD$for each child after the 5th child.	2015	III	https://www.msf.gov.sg/policies/Strong-and-Stable-Families/Supporting-Families/Pages/Baby-Bonus-Scheme.aspx
	Singapore has established child Development Accounts (CAD), which are special savings accounts for children and can be used for approved purposes. The CDA of eligible children born on or after March 24, 2016 or meeting the expected delivery date has been increased and when the CDA is opened, the child will receive a CDA benefit of $3000SGD.	-	-	https://www.msf.gov.sg/policies/Strong-and-Stable-Families/Supporting-Families/Pages/Baby-Bonus-Scheme.aspx#

	Infertility diagnosis and treatment leave	Women undergoing assisted conception procedures (ACP) are entitled to hospital leave	-	-	https://www.moh.gov.sg/news-highlights/details/response-to-adjournment-motion-on-providing-more-help-for-those-undergoing-ivf
		The Employment Act (Amendment) limits private paid maternity leave to the first two children and limits paid maternity leave to eight weeks	1973	II	A Comparative Study of Fertility Policies in China and Singapore
		If the child is a Singaporean citizen, the paid maternity leave under the Joint Savings for Child Development Act is 16 weeks.	2013	II	https://sso.agc.gov.sg/Act/CDCSA2001#pr9-
		If the child is not a Singaporean citizen, the paid maternity leave under the Employment Act is 12 weeks.	2014	II	https://sso.agc.gov.sg/Act/EmA1968#P1IX-
Social security	Parental leave security	If children are Singapore citizens, under the Joint Savings for Child Development Act, parents of children under the age of seven are entitled to six days of child care leave per year, two days for those between the ages of seven and 12, and up to four weeks of unpaid leave for unforeseen circumstances.	2013	II	https://sso.agc.gov.sg/Act/CDCSA2001#P1III-
		Children who are not Singapore citizens are entitled to 2 days parental leave under the Employment Act	2014	II	https://sso.agc.gov.sg/Act/EmA1968#P1IX-
		Fathers are entitled to two weeks' paid paternity leave provided by the government	2017	III	https://www.mom.gov.sg/employment-practices/leave/paternity-leave
		If the child is a Singapore citizen and is born on or after 1 July 2017 or is expected to be delivered after 1 July 2017, the father may apply to share up to four weeks of his wife's 16 weeks of government-paid maternity leave as parental leave	2017	III	https://www.mom.gov.sg/employment-practices/leave/shared-parental-leave
	Childcare and educational security	Starting 1990, a tax rebate of 20,000 SGD (US$18,000 in 2010 dollars, factoring historic exchange rates) were given to mothers who had their second child before the age of 28	1990	II	https://en.wikipedia.org/wiki/Population_planning_in_Singapore
		Singapore government provides a subsidy for babies from 2 months to 18 months, which needs to be reviewed by EDCA before the subsidy is issued. If the parents are working, the basic subsidy can	-	-	https://www.ecda.gov.sg/Pages/Subsidies-and-Financial-Assistance.aspx

	be $600SGD, and the additional subsidy can reach up to $710SGD. If the parents are not working, a basic allowance of $150SGD is available and no additional allowance is available. Meet some special circumstances can be approved to obtain higher subsidies.			
	The Singapore government provides subsidies for children aged 18 months to 6 years, which should be reviewed by EDCA before the payment is made. If the parents are working, the basic subsidy can be 300SGD$, with additional subsidies up to 465SGD$; If the parents are not working, a basic allowance of $150SGD is available and no additional allowance is available. Meet some special circumstances can be approved to obtain higher subsidies.	-	-	https://www.ecda.gov.sg/Pages/Subsidies-and-Financial-Assistance.aspx
	Low-income families with difficult family circumstances may apply for financial assistance if they find it difficult to pay for child care after receiving basic subsidies and additional subsidies.	-	-	https://www.ecda.gov.sg/Pages/Subsidies-and-Financial-Assistance.aspx
	Singapore implements the Kindergarten Financial Assistance Scheme (KiFAS). The payment of the subsidy is means-tested. Low-income families can receive the subsidy after verification, and the subsidy is directly paid to the kindergarten where the child attends.	-	-	https://www.ecda.gov.sg/Pages/Subsidies-and-Financial-Assistance.aspx#KIFAS
	Singapore's Ministry of Social and Family Development (MSF) has set up a number of Childcare Service Delivery points (PSP) to provide childcare support services on a regional basis. PSP is a one-stop service provider that works closely with schools in designated districts to provide evidence-based parenting programs and follow-up counselling support for parents who need it.	2019	III	https://www.msf.gov.sg/policies/Strong-and-Stable-Families/Supporting-Families/Pages/Parenting.aspx
	The government has revised the Income tax Bill to cut taxes by 750 SGD$ for a single child, 500 SGD$ for a second child and 500 SGD$ for a third child	1972	II	A Comparative Study of Fertility Policies in China and Singapore-YaoDan
Life security	The main measures are as follows: In terms of children's enrollment, female children with high education have greater priority in choosing schools; In terms of tax credits, the more educated parents are, the higher the tax credits for their children	1984	II	A Comparative Study of Fertility Policies in China and Singapore
	Implement tax cuts for families with three or more children	1987	II	Implications of The International Population Policy Shift for China

		The Assisted Reproduction Program (ARP) insurance plan protects against unexpected newborn care costs for babies caused by in vitro fertilization (IVF) in cases where special care is required. If the baby is admitted to an intensive care nursery or a neonatal intensive care unit (NICU), they receive cash benefits of $150 or $300 per day, respectively. Since 1994, couples have had to take out ARP insurance when they join IVF because MediShield did not previously cover newborn diseases.	1994	II	https://www.moh.gov.sg/news-highlights/details/insurance-for-in-vitro-fertilisation
		The MediSave maternity kit covers delivery costs in both public and private hospitals, as well as pre-delivery medical care, such as counselling and ultrasound.	-	-	https://www.moh.gov.sg/cost-financing/healthcare-schemes-subsidies/marriage-and-parenthood-schemes
		MediSave Newborn Benefit (MGN) can also be used to offset hospitalization costs, with a daily MediSave withdrawal limit of $450	2012	II	https://www.moh.gov.sg/news-highlights/details/insurance-for-in-vitro-fertilisation
Service Package	Maternal and child health services	MediShield Life, which provides universal coverage for all Singaporean citizens from birth, provides up to S $700 per day for general ward hospitalization and S $1,200 per day for intensive care unit hospitalization.	-	-	https://www.moh.gov.sg/news-highlights/details/insurance-for-in-vitro-fertilisation
		MediShield Life's coverage extends to severe pregnancy or childbirth complications	2019	III	https://www.moh.gov.sg/news-highlights/details/insurance-for-in-vitro-fertilisation
		When a child is born as a Singaporean citizen, a Medisave account will be automatically created in their name upon birth registration and a Medisave grant of $4,000 will be deposited. The grant can be used to cover the child's medical care costs, such as MediShield premiums, child vaccinations in the recommended community, hospitalization and approved outpatient treatment.	-	-	https://www.moh.gov.sg/cost-financing/healthcare-schemes-subsidies/marriage-and-parenthood-schemes
		The Singapore government subsidizes CDS for Singaporean children who participate in the Child Development Screening (CDS)	-	-	https://www.moh.gov.sg/cost-financing/healthcare-schemes-subsidies/vaccination-and-

		The Singapore Government subsidizes vaccination for Singaporean children who qualify for the National Childhood Immunisation Programme (NCIS).	-	-	childhood-developmental-screening-subsidies https://www.moh.gov.sg/cost-financing/healthcare-schemes-subsidies/vaccination-and-childhood-developmental-screening-subsidies
Technology development guarantee	Assisted reproductive technology development	From 1 January 2020, the Ministry of Health removed the age limit of 45 years for women to receive ART treatment and the upper limit on the number of times all women can receive ART treatment	2020	II	https://www.moh.gov.sg/news-highlights/details/more-support-for-childhood-preventive-healthcare-and-fertility-treatments
Reproductive health promotion	Reproductive health education and protection	The Ministry of Education in Singapore has developed a tripartite framework for sex education, including family, school and society, and has promulgated sex education programmes that meet the needs of different age groups, namely the Growing Years Programme and the Empowering Youth Programme. Under the plan, Singaporean students between the ages of 11 and 18 nationwide can receive sex education for between two and five hours a year, depending on the age.	2000	II	A Study on Sex Education Programmes in Singapore

H. South Korea

Dimension	Index	Policies	Year	Grade	Source
		The South Korean government provided economic security for low-income infertility population.	2006	II	http://world.people.com.cn/n/2013/1017/c1002-23240293.html
	Insurance coverage	The Infertile Couple Support Policy was extended so that all types of ARTs are covered by the national health insurance.	2017	III	Reproductive technologies as population control: how pronatalist policies harm reproductive health in South Korea
		The number of subsidies for maternity medical expenses increased, which are used by pregnant women to pay for hospital examinations.	2021	III	http://www.legaldaily.com.cn/index/content/2020-12/21/content_8386900.htm
	Financial assistance	The government provided subsidies for infertile patients.	2006	II	http://world.people.com.cn/n/2013/1017/c1002-23240293.html
Economic Support		The baby bonus is 200000 KRW for newborns and 150000 KRW for one-year-old children.	2006	II	https://elaw.klri.re.kr/eng_service/lawView.do?hseq=25680&lang=ENG#:~:text=The%20purpose%20of%20this%20Act,cope%20with%20changes%20following%20low
	Economic reward	Financial support for childbirth were increasing during 2011-2015.	2011	III	https://elaw.klri.re.kr/eng_service/lawView.do?hseq=25680&lang=ENG#:~:text=The%20purpose%20of%20this%20Act,cope%20with%20changes%20following%20low
		The amount of baby bonus was increased, additional 2 million KRW were provided for puerpera.	2021	III	https://elaw.klri.re.kr/eng_service/lawView.do?hseq=25680&lang=ENG#:~:text=The%20purpose%20of%20this%20Act,cope%20with%20changes%20following%20low

	Infertility diagnosis and treatment leave security	A three-day leave for diagnosis and treatment of infertility was granted.	2017	II	https://elaw.klri.re.kr/eng_service/lawView.do?hseq=25680&lang=ENG#:~:text=The%20purpose%20of%20this%20Act,cope%20with%20changes%20following%20low
		Women should get pay raises before and after maternity, and the coverage of maternity and paternity leave was expanded.	2006	II	https://elaw.klri.re.kr/eng_service/lawView.do?hseq=25680&lang=ENG#:~:text=The%20purpose%20of%20this%20Act,cope%20with%20changes%20following%20low
Social security	Parental leave security	Flexible working hours were tried out in government sectors.	2010	II	http://news.xinhuanet.com/world/2010-12/18/c_12893783.htm
		In South Korea, paid maternity leave was 90 days.	-	-	http://chinese.joins.com/news/articleView.html?idxno=48169
		Under the new "3+3" parental leave system, couples who take parental leave in the first 12 months of their child's birth could enjoy up to three months of parental leave and up to 3 million KRW per person per month.	2021	II	http://www.legaldaily.com.cn/index/content/2020-12/21/content_8386900.htm
		To protect pregnant women at work, punitive damages were introduced.	2021	II	http://www.legaldaily.com.cn/index/content/2020-12/21/content_8386900.htm
	Child care and education security	For the purpose of reducing childcare costs financial support for childcare and pre-school costs were increased, the frustructure of childcare facilities were expanded, the availability of childcare facilities for professionals were increased, and the "after school" program to reduce childcare costs were expanded.	2006	II	https://elaw.klri.re.kr/eng_service/lawView.do?hseq=25680&lang=ENG#:~:text=The%20purpose%20of%20this%20Act,cope%20with%20changes%20following%20low

	Creating an environment conducive to child-rearing, which helps to relief the burden of child-rearing for families.	2011	II	https://elaw.klri.re.kr/eng_service/lawView.do?hseq=25680&lang=ENG#:~:text=The%20purpose%20of%20this%20Act,cope%20with%20changes%20following%20low
	Since 2013, all families with children between the ages of 0 and 5 in Korea have been entitled to nursery care fees or a family care allowance (200,000 KRW).	2013	II	http://chinese.joins.com
	Guarantee of high-quality conservation services: The government has introduced an evaluation and certification system to improve the quality of private conservation services	-	-	http://chinese.joins.com/news/articleView.html?idxno=48169
	Financial support for childcare costs were increased and Education reform has been carried out to reduce the cost of private education.	2016	II	https://elaw.klri.re.kr/eng_service/lawView.do?hseq=25680&lang=ENG#:~:text=The%20purpose%20of%20this%20Act,cope%20with%20changes%20following%20low
	Aiming at families with many children, preferential tax system has been introduced.	2006	II	https://elaw.klri.re.kr/eng_service/lawView.do?hseq=25680&lang=ENG#:~:text=The%20purpose%20of%20this%20Act,cope%20with%20changes%20following%20low
Life security	Housing assistance has been provided to 50,000 households per year for newly married couples who did not own a home and were low-income (defined as less than 70 percent of the average income of urban workers in the previous year and less than 100 percent for dual-income couples).	2006	II	http://chinese.joins.com/news/articleView.html?idxno=48169
	Low interest rate home loans were offered.	2016	II	https://elaw.klri.re.kr/eng_service/lawView.do?hseq=25680&lang=ENG#:~:text=The%20purpose%20of%20this%20Act

					,cope%20with%20changes%20following%20low
		Providing support for families with multiple children (private public rental housing, full tuition subsidy for three children, etc.)	2021	II	http://www.legaldaily.com.cn/index/content/2020-12/21/content_8386900.htm
Service Package	Maternal and child health service	Law on Maternal and Child Health	1973	II	https://elaw.klri.re.kr/eng_mobile/viewer.do?hseq=33648&type=part&key=38
		Medical assistance was provided for newborn babies	2006	II	http://chinese.joins.com/news/articleView.html?idxno=48169
		Nutrition guidance was given to pregnant women and newborns.	2006	II	http://chinese.joins.com/news/articleView.html?idxno=48169
		State subsidies were provided for the vaccination of newborns	2006	II	http://chinese.joins.com/news/articleView.html?idxno=48169
Technology development guarantee	Assisted reproductive technology development	In 2019, the mayor of Seoul pledged financial support to expand the number of public health centers offering assisted reproductive treatment and to provide mental health services through public advice. At the same time, cooperation with the central government was made to actively solve this problem.	2019	II	https://www.centreforpublicimpact.org/case-study/access-fertility-treatment-democracy-seoul/
Reproductive health promotion	Reproductive health education and protection	Public health centres provided sex education and sexually transmitted disease prevention	2011	II	History of Syphilis and Gonorrhea in Korea. Infect Chemother.
		The South Korean government issued STD guidelines for South Korea in 2011 (updated in 2016)	2011	II	History of Syphilis and Gonorrhea in Korea. Infect Chemother.

I. the United States

Dimension	Index	Policies	Year	Grade	Source
Economic Support	Insurance coverage	Since 1985, 19 states in America have passed fertility insurance coverage laws, 13 of those laws include IVF coverage, and 11 states have fertility preservation laws for iatrogenic (medically-induced) infertility. Summaries of state fertility insurance laws were as shown as Table S7 (see below).	1985	II	https://resolve.org/what-are-my-options/insurance-coverage/infertility-coverage-state/
	Financial assistance	-	-	-	-
	Economic reward	-	-	-	-
Social security	Infertility diagnosis and treatment leave security	-	-	-	-
	Parental leave security	The Pregnancy Discrimination Act (PDA) requires that all employers treat employees who are mothers of newborns in the same way as other workers who are physically unable to work. Female employees who have recovered from childbirth shall be allowed to restore their pre-birth conditions. Work position; the disability insurance policy that provides part of the salary for employees during vacations must also cover "pregnancy-related disability."	1978	II	Research on Support Policy for Parenting Parents in the United States-Parenting Holidays and ECEC Services
		The 12-week Unpaid Employment-protected Leave provided by the Family and Medical Leave Act (FMLA) includes “parental care leave” and care for parents of newborns or newly adopted children in need of care "Family Care Leave" for close relatives or family members with severe health	1993	III	Research on Support Policy for Parenting Parents in the United States-Parenting Holidays and ECEC Services

	conditions.<br>This holiday does not include employees of companies with fewer than 50 employees, employees who have worked in the current company for less than a year, or employees who work less than 24 hours a week.			
	California implements Paid Family Leave (PFL), which can provide a 6-week income guarantee for families who need to take care of young children or sick elderly	2004	III	Research on Support Policy for Parenting Parents in the United States-Parenting Holidays and ECEC Services
	New Jersey implements paid family leave (PFL), which can provide 6 weeks of income security for families who need to take care of young children or sick elderly	2009	III	Research on Support Policy for Parenting Parents in the United States-Parenting Holidays and ECEC Services
	Rhode Island implements Paid Family Leave (PFL), which can provide a four-week income guarantee for families who need to take care of young children or sick elderly people.	2014	III	Research on Support Policy for Parenting Parents in the United States-Parenting Holidays and ECEC Services
	NJ workers can now get up to 12 consecutive weeks of benefits per year to bond with a new child or care for a loved one. NJ workers can now get up to 85% of their average weekly wage.	2020	III	https://myleavebenefits.nj.gov/worker/resources/Bonding_expansion_2.shtml
	California and New York implement short-term disability insurance plans (SDI), and Hawaii, New Jersey, Rhode Island, and Puerto Rico implement temporary disability insurance plans (TDI).<br>These two plans cover emergency situations outside the workplace, including incapacity to work, and provide 6 to 10 weeks of income support for disabled mothers during childbirth and childbirth through TDI.	-	-	Research on Support Policy for Parenting Parents in the United States-Parenting Holidays and ECEC Services
Child care and education security	ECEC policy covers children from birth until reaching the age designated by the state for compulsory education.<br>The age of compulsory education is determined by each state and ranges from five to eight years old.	-	-	Research on Support Policy for Parenting Parents in the United States-Parenting Holidays and ECEC Services

		The institutions give direct and indirect financial subsidies, such as grants, contracts and tax incentives. Temporary Assistance for Poor Families (TANF) is determined by the "Personal Responsibility and Work Opportunity Coordination Act". It aims to provide assistance to poor families with children. It is an important public assistance program in the United States. The sources of relief funds include both federal funding to the states and supporting funds from the states.	1996	II	Research on Support Policy for Parenting Parents in the United States-Parenting Holidays and ECEC Services
	Life security	Under the law, if a plan includes children, a parent can cover children on their health insurance plan until the child turns 26 years old.	2010	II	https://www.cms.gov/CCIIO/Programs-and-Initiatives/Health-Insurance-Market-Reforms
		Under those rules, which were established by 2017's Tax Cuts and Jobs Act (TCJA), taxpayers could claim a CTC of up to $2,000 for each child under age 17. The credit would decrease by 5 percent of adjusted gross income over $200,000 for single parents ($400,000 for married couples). If the credit exceeded taxes owed, taxpayers could receive up to $1,400 as a tax refund known as the additional child tax credit (ACTC) or refundable CTC. However, under the TCJA rules, the ACTC would be limited to 15 percent of earnings above $2,500	-	-	https://www.taxpolicycenter.org/briefing-book/what-child-tax-credit
Service Package	Maternal and child health services	Group health plans and health insurance companies that are subject to the Newborn and Maternal Health Protection Act (NMHPA) must not limit the hospitalization costs associated with childbirth to within 48 hours after vaginal delivery or within 96 hours after cesarean delivery.	1996	II	-
Technology developmen t guarantee	Assisted reproduct ive technolog y	-	-	-	https://www.kff.org/womens-health-policy/issue-brief/coverage-and-use-of-fertility-services-in-the-u-s/

	development				
Reproductive health promotion	Reproductive health education and protection	Sexually transmitted disease services: sexually transmitted infection (STI) and human immunodeficiency virus (HIV) prevention education, counseling, testing and referral	2014	II	https://www.cdc.gov/mmWr/preview/mmwrhtml/rr6304a1.htm
		The Title X program implemented by HRSA and OASH screens young women for chlamydia. The planned number of services in 2015 and 2016 was 1,155,500 and 1,195,000, respectively.	2015	III	https://www.hhs.gov/about/budget/performance/goal-1-objective-e/index.html

Table S7. Summaries of state fertility insurance laws

**States**	**Fertility insurance coverage laws**	**Laws included IVF coverage**	**Fertility preservation laws for iatrogenic (medically-induced) infertility**
Arkansas	√	√	
California	√		√
Colorado	√	√	√
Connecticut	√	√	√
Delaware	√	√	√
Hawaii	√	√	
Illinois	√	√	√
Louisiana	√		
Maryland	√	√	√
Massachusetts	√	√	
Montana	√		
New Hampshire	√	√	√
New Jersey	√	√	√
New York State	√	√	√
Ohio	√		
Rhode Island	√	√	√
Texas	√	√	√
Utah	√	√	
West Virginia	√		

J. United Kingdom

Dimension	Index	Policies	Year	Grade	Source
Economic Support	Insurance coverage	Health insurance policies usually do not cover IVF or fertility treatments, and insurance companies will not cover any fertility treatments such as IVF.	2016	I	https://boughtbymany.com/news/article/private-health-insurance-pregnancy-uk/
		In most cases you should be able to have some fertility testing on the NHS. This includes some areas that don't offer any funded fertility treatment at all.	-	-	https://www.hfea.gov.uk/treatments/explore-all-treatments/costs-and-funding/
		If you live in England, it gets more complicated. Decisions about who can have fertility treatment (and how much) are decided locally by Clinical Commissioning Groups (CCGs). So, in some parts of the country, you may be able to get three attempts at IVF whilst in another part of the country you would only be able to get one attempt at IVF (or even none).	2017	II	https://www.hfea.gov.uk/treatments/explore-all-treatments/costs-and-funding/
	Financial assistance	When receiving fertility treatment, low-income people can get free prescription drugs after applying for the HC2 certificate	-	-	https://www.nhs.uk/nhs-services/prescriptions-and-pharmacies/who-can-get-free-prescriptions/
	Economic reward	For the first child of a woman who has received means-tested social relief benefits, a maternity subsidy of £500 will be given.	-	-	Research on the Childbearing Security System Abroad and Its Enlightenment to our country
Social security	Infertility diagnosis and treatment leave security	-	-	-	-
	Parental leave security	British women are entitled to 18 weeks of maternity leave according to law	1999	II	https://www.legislation.gov.uk/uksi/1999/3312/regulation/7
		British women are entitled to 26 weeks of maternity leave according to law	2002	II	https://www.legislation.gov.uk/uksi/2002/2789/regulation/8/made

Maternity leave is 52 weeks, but only 39 weeks of paid leave are granted.<br>In the first 6 weeks, the disbursement amount is 90% of the income, and there is no payment cap; in the remaining 33 weeks, 90% of the income is paid every week, up to a maximum of £139.58 per week.<br>During the statutory maternity leave, women's employment rights are protected.	2015	II	Research on the Childbearing Security System Abroad and Its Enlightenment to our country
Working men can enjoy it during the spouse's childbirth period. The enjoyment time is 2 weeks, and the treatment payment level is 90% of the income, and the maximum is 139.58 pounds per week.	2015	III	https://www.gov.uk/shared-parental-leave-and-pay/what-youll-get,https://www.legislation.gov.uk/nisr/2015/93/contents
The parental leave is 18 weeks and no allowance is paid.	2015	III	Research on the Childbearing Security System Abroad and Its Enlightenment to our country
For self-employed and unemployed employees who are not entitled to paid maternity leave, they can enjoy 39 weeks of maternity allowance. The standard of treatment is £139.58 per week. If the weekly salary is less than this amount, 90% of the salary is paid.<br>The prerequisite is that the self-employed person pays 13 weeks to 66 weeks of national insurance, the employee or self-employed person needs to work for at least 26 weeks, and at least 13 weeks of weekly wages are not less than 30 pounds.	-	-	https://www.gov.uk/maternity-allowance/what-youll-get
For non-working residents, they can enjoy 14 weeks of maternity allowance, and the standard of treatment is 27 pounds per week.	-	-	https://www.gov.uk/maternity-allowance/what-youll-get
In addition to statutory paid leave, the UK also has statutory unpaid parental leave, that is, every parent can take unpaid parental leave to look after their children under 5 years old.<br>If there are disabled children in the family, the age is relaxed to 18 years old.<br>Taking parental leave requires the applicant to have worked in this job continuously for at least one year.	-	-	Analysis of Foreign Family Policies Affecting Fertility

	Fathers and mothers can take a total of no more than 13 weeks of parental leave for each child under 5 years old.			
	For low-income families, they can use the government's "income subsidy" policy to get a certain amount of financial assistance when taking parental leave.	-	-	Analysis of Foreign Family Policies Affecting Fertility
	The British government has increased its funding for the Early Childhood Education and Conservation Project (ECEC). It also provides support to families using nanny and family custody fees through the custody fee part of the work tax credit and employer-sponsored custody fee vouchers.	1990	II	Return of the Nanny: Public Policy towards In-home Childcare in the UK, Canada and Australia
	Compulsory education is required for children aged 5-16, and the curriculum will be unified nationwide at this stage.	1998	II	The Evolution and Enlightenment of British Compulsory Education Regulations
Child care and education security	The United Kingdom promulgated the Green Paper "Responding to Childcare Challenges", also known as "The National Childcare Strategy for England".<br>The Green Paper proposes a "three-step" action plan. The main contents include improving the quality of childcare; helping working families reduce the burden of childcare through taxation and preferential systems; expanding childcare venues and improving relevant information.	1998	II	The experience of childcare services for infants and children aged 0-3 in the UK and its enlightenment to my country
	The British government requires local governments to list early education funds separately and introduce a "Single Funding Formula".<br>The British central government not only directly allocates funds to kindergarten institutions, but also issues additional special funds to institutions through local governments.	2007	III	The experience of childcare services for infants and children aged 0-4 in the UK and its enlightenment to my country
	The United Kingdom introduced the Statutory Framework For The Early Years Foundation Stage (EYFS), which treats the care and education of children aged 0-5 as a unified whole, and addresses children's learning and development requirements, development assessment, and safety<br>And health requirements, etc.	2008	III	The experience of childcare services for infants and children aged 0-5 in the UK and its enlightenment to my country

	All childcare institutions in the UK must be registered with the government, which sets a barrier to entry for childcare institutions. Secondly, the government also stipulates that nursery service practitioners must register, must have a nursery qualification certificate issued by the regulatory authority, and require them to participate in training organized by the local competent authority within six months after registration; family nursery practitioners must have A minimum level 2 qualification certificate related to the field of infant care.	-	-	Return of the Nanny: Public Policy towards In-home Childcare in the UK, Canada and Australia
	The National Education Standards Bureau provides financial support for 0–5-year-old child care and education, formulates unified standards, sets integrated courses, conducts teacher certification, and supervises and manages all certified early education institutions. The family welfare department, labor and Training departments, health and sanitation departments, etc. participate in standard setting, teacher recruitment, guidance on site selection and other links to jointly ensure the quality of childcare services.	-	-	The experience of childcare services for infants and children aged 0-3 in the UK and its enlightenment to my country
	The maternity medical treatment enjoyed by the nationals is covered by the medical insurance. Citizens can enjoy the National Health Service (NHS) established by the British government for all citizens without paying fees. Citizens need to pay registration fees and a small number of items that need to be paid by themselves; other expenses are paid by the public finances.	1946	II	Research on the Childbearing Security System Abroad and Its Enlightenment to our country
Life security	Parents with children under the age of 16 or under 20 who are attending full-time school in a state-recognized educational institution can apply for the “Children’s Tax Credit” program	-	-	https://www.gov.uk/child-tax-credit
	If the family has children under the age of 16 (or under the age of 20 if they receive state-approved education or training), they are usually eligible for child benefits. Child benefits are usually paid every 4 weeks on Monday or Tuesday. The funds for this government subsidy are not taxed, and the eligibility for application is not restricted by the amount of personal income and deposits.	-	-	https://www.gov.uk/child-benefit

		There are differences in parity in the amount of subsidy.<br>The first child or only child will get £20.30 a week, and all other children will get support at £13.40 a week.<br>If the child is under the age of 12 and the dependent does not have a job or has insufficient income to pay the national insurance contributions, the child welfare can provide the dependent with a national insurance credit.<br>These credits are included in the dependent's state pension.	-	-	https://www.gov.uk/child-benefit
		Parents can establish "Children's Trust Fund" accounts for their children.<br>When each eligible child establishes a new account, the government provides an incentive fund of up to £250, after which family members and even relatives and friends can deposit a tax-free £3,720 into this account every year.	-	-	https://www.gov.uk/child-trust-funds
Service Package	Maternal and child health service	You can get free NHS prescriptions if you are pregnant or have had a baby in the previous 12 months and have a valid maternity exemption certificate (MatEx)	-	-	https://www.nhs.uk/nhs-services/prescriptions-and-pharmacies/who-can-get-free-prescriptions/
		You can book an appointment with your GP or directly with your midwife and start antenatal care. If you're pregnant in England you will be offered:2 pregnancy ultrasound scans at 8 to 14 weeks and 18 to 21 weeks, antenatal screening tests to find out the chance of your baby having certain conditions, such as Down's syndrome, blood tests to check for syphilis, HIV and hepatitis B, screening for sickle cell and thalassemia.	-	-	https://www.nhs.uk/pregnancy/your-pregnancy-care/your-antenatal-care/
		The NHS will provide free prenatal courses for pregnant women, including taking care of and feeding their babies, maintaining health during pregnancy, and formulating birth plans.	-	-	https://www.nhs.uk/pregnancy/your-pregnancy-care/your-antenatal-care/
		The NHS provides free prenatal consultation, which is provided by a midwife or doctor, who will provide psychological and social support for pregnant women	-	-	https://www.nhs.uk/pregnancy/your-pregnancy-care/your-antenatal-care/
		Children are vaccinated at your GP surgery or a local child health clinic, and services are provided free of charge by the NHS.	-	-	https://www.nhs.uk/conditions/vaccinations/booking-your-childs-vaccination-appointment/

Technology development guarantee	Assisted reproductive technology development	The Scottish government stipulates that if there are children in the family, but one of the spouses has no biological children, IVF treatment can also be provided for the family.	2016	II	http://www.fertilityfairness.co.uk/scotland-expands-access-to-ivf-treatment/
		The Competition and Market Administration (CMA) drafted the "Draft Guidelines for Consumer Law for Fertility Clinics", which will help patients understand assisted reproductive methods, strengthen the compliance of fertility clinics with consumer laws, and should help resolve issues identified in the industry. some problems.	2020	II	https://www.gov.uk/government/consultations/draft-guidance-for-fertility-clinics-on-consumer-law
Reproductive health promotion	Reproductive health education and protection	In the "Education Act" UK is further proposed that all public schools must teach content related to sex education, such as the physiological development of adolescence, reproduction and the spread of viruses. High schools must also teach about HIV and sexually transmitted diseases.	1996	II	An Analysis of Sex Education in the National Curriculum System of British Schools
		The National Children's Bureau issued the "Sex and Gender Relations Education Framework", which is the first educational framework specifically promulgated for sex education in the UK.	2003	II	An Analysis of Sex Education in the National Curriculum System of British Schools
		The official national institution, the Sex Education Forum launched a "Sex Education Matters" campaign, which reaffirmed the importance of sex education	2012	II	An Analysis of Sex Education in the National Curriculum System of British Schools
		The NHS provides free sexual health services, which can be used by everyone regardless of their gender, age, ethnic origin and sexual orientation. The services provided include contraception and sexually transmitted disease prevention services, pregnancy services, abortion services, sexual counseling, Sexual assault services, etc.	2018	III	https://www.nhs.uk/nhs-services/sexual-health-services/guide-to-sexual-health-services/

Appendix 3. Calculation process of CIIs

A. Australia

Dimension	Index	Stage I	Stage II	Stage III	Total score	Score of Infertility-related policies	Average score of ESSTR	CIIs
Economic support	Insurance coverage	2	12	14	40	71	57	31
	Financial assistance	NA	NA	NA	NA	NA		
	Economic reward	14	4	10	24	43		
Social security	Infertility diagnosis and treatment leave security	28	0	0	0	0	14	
	Parental leave security	22	1	5	11	20		
	Child care and education security	16	12	0	12	21		
	Life security	NA	NA	NA	NA	NA		
Service package	Maternal and child health service	18	10	0	10	18	18	
Technology development guarantee	Assisted reproductive technology development	0	28	0	28	50	50	
Reproductive health protection	Reproductive health education and protection	20	8	0	8	14	14	

B. Canada

Dimension	Index	Stage I	Stage II	Stage III	Total score	Score of Infertility-related policies	Average score of ESSTR	CIIs
Economic support	Insurance coverage	20	8	0	8	14	37	41
	Financial assistance	NA	NA	NA	NA	NA		
	Economic reward	0	23	5	33	59		
Social security	Infertility diagnosis and treatment leave security	NA	NA	NA	NA	NA	57	
	Parental leave security	0	28	0	28	50		
	Child care and education security	8	8	12	32	57		
	Life security	0	20	8	36	64		
Service package	Maternal and child health service	0	28	0	28	50	50	
Technology development guarantee	Assisted reproductive technology development	NA	NA	NA	NA	NA	NA	
Reproductive health protection	Reproductive health education and protection	16	12	0	12	21	21	

C. China

Dimension	Index	Stage I	Stage II	Stage III	Total score	Score of Infertility-related policies	Average score of ESSTR	CIIs
Economic support	Insurance coverage	28	0	0	0	0	7	19
	Financial assistance	23	5	0	5	9		
	Economic reward	21	7	0	7	13		
Social security	Infertility diagnosis and treatment leave security	28	0	0	0	0	12	
	Parental leave security	21	7	0	7	13		
	Child care and education security	20	8	0	8	14		
	Life security	16	12	0	12	21		
Service package	Maternal and child health service	5	22	1	24	43	43	
Technology development guarantee	Assisted reproductive technology development	17	11	0	11	20	20	
Reproductive health protection	Reproductive health education and protection	21	6	1	8	14	14	

D. France

Dimension	Index	Stage I	Stage II	Stage III	Total score	Score of Infertility-related policies	Average score of ESSTR	CIIs
Economic support	Insurance coverage	NA	NA	NA	NA	NA	50	47
	Financial assistance	NA	NA	NA	NA	NA		
	Economic reward	0	28	0	28	50		
Social security	Infertility diagnosis and treatment leave security	NA	NA	NA	NA	NA	86	
	Parental leave security	0	12	16	44	79		
	Child care and education security	0	4	24	52	93		
	Life security	0	7	21	49	88		
Service package	Maternal and child health service	NA	NA	NA	NA	NA	NA	
Technology development guarantee	Assisted reproductive technology development	28	0	0	0	0	0	
Reproductive health protection	Reproductive health education and protection	0	28	0	28	50	50	

E. India

Dimension	Index	Stage I	Stage II	Stage III	Total score	Score of Infertility-related policies	Average score of ESSTR	CIIs
Economic support	Insurance coverage	28	0	0	0	0	20	13
	Financial assistance	18	10	0	10	18		
	Economic reward	5	23	0	23	41		
Social security	Infertility diagnosis and treatment leave security	NA	NA	NA	NA	NA	31	
	Parental leave security	0	28	0	28	50		
	Child care and education security	27	1	0	1	2		
	Life security	5	23	0	23	41		
Service package	Maternal and child health service	28	0	0	0	0	0	
Technology development guarantee	Assisted reproductive technology development	27	1	0	1	2	2	
Reproductive health protection	Reproductive health education and protection	NA	NA	NA	NA	NA	NA	

F. Japan

Dimension	Index	Stage I	Stage II	Stage III	Total score	Score of Infertility-related policies	Average score of ESSTR	CIIs
Economic support	Insurance coverage	28	0	0	0	0	23	52
	Financial assistance	4	22	2	26	46		
	Economic reward	NA	NA	NA	NA	NA		
Social security	Infertility diagnosis and treatment leave security	27	1	0	1	2	37	
	Parental leave security	1	4	23	50	89		
	Child care and education security	25	3	0	3	5		
	Life security	0	28	0	28	50		
Service package	Maternal and child health service	0	28	0	28	50	50	
Technology development guarantee	Assisted reproductive technology development	NA	NA	NA	NA	NA	NA	
Reproductive health protection	Reproductive health education and protection	0	2	26	54	96	96	

G. Singapore

Dimension	Index	Stage I	Stage II	Stage III	Total score	Score of Infertility-related policies	Average score of ESSTR	CIIs
Economic support	Insurance coverage	0	23	5	33	59	48	35
	Financial assistance	NA	NA	NA	NA	NA		
	Economic reward	10	15	3	21	38		
Social security	Infertility diagnosis and treatment leave security	NA	NA	NA	NA	NA	51	
	Parental leave security	0	27	1	29	52		
	Child care and education security	0	28	0	28	50		
	Life security	0	28	0	28	50		
Service package	Maternal and child health service	4	24	0	24	43	43	
Technology development guarantee	Assisted reproductive technology development	28	0	0	0	0	0	
Reproductive health protection	Reproductive health education and protection	10	18	0	18	32	32	

H. South Korea

Dimension	Index	Stage I	Stage II	Stage III	Total score	Score of Infertility-related policies	Average score of ESSTR	CIIs
Economic support	Insurance coverage	16	11	1	13	23	26	21
	Financial assistance	16	12	0	12	21		
	Economic reward	16	5	7	19	34		
Social security	Infertility diagnosis and treatment leave security	27	1	0	1	2	17	
	Parental leave security	16	12	0	12	21		
	Child care and education security	16	12	0	12	21		
	Life security	16	12	0	12	21		
Service package	Maternal and child health service	0	28	0	28	50	50	
Technology development guarantee	Assisted reproductive technology development	28	0	0	0	0	0	
Reproductive health protection	Reproductive health education and protection	21	7	0	7	13	13	

I. the United States

Dimension	Index	Stage I	Stage II	Stage III	Total score	Score of Infertility-related policies	Average score of ESSTR	CIIs
Economic support	Insurance coverage	0	28	0	28	50	50	42
	Financial assistance	NA	NA	NA	NA	NA		
	Economic reward	NA	NA	NA	NA	NA		
Social security	Infertility diagnosis and treatment leave security	NA	NA	NA	NA	NA	67	
	Parental leave security	0	3	25	53	95		
	Child care and education security	NA	NA	NA	NA	NA		
	Life security	6	22	0	22	39		
Service package	Maternal and child health service	6	22	0	22	39	39	
Technology development guarantee	Assisted reproductive technology development	NA	NA	NA	NA	NA	NA	
Reproductive health protection	Reproductive health education and protection	24	1	3	7	13	13	

J. United Kingdom

Dimension	Index	Stage I	Stage II	Stage III	Total score	Score of Infertility-related policies	Average score of ESSTR	CIIs
Economic support	Insurance coverage	27	1	0	1	2	2	23
	Financial assistance	NA	NA	NA	NA	NA		
	Economic reward	NA	NA	NA	NA	NA		
Social security	Infertility diagnosis and treatment leave security	NA	NA	NA	NA	NA	48	
	Parental leave security	9	16	3	22	39		
	Child care and education security	8	9	11	31	55		
	Life security	0	28	0	28	50		
Service package	Maternal and child health service	NA	NA	NA	NA	NA	NA	
Technology development guarantee	Assisted reproductive technology development	26	2	0	2	4	4	
Reproductive health protection	Reproductive health education and protection	6	22	0	22	39	39	
